# Enhancer Chip: Detecting Human Copy Number Variations in Regulatory Elements

**DOI:** 10.1371/journal.pone.0052264

**Published:** 2012-12-20

**Authors:** Marco Savarese, Giulio Piluso, Daniela Orteschi, Giuseppina Di Fruscio, Manuela Dionisi, Francesca del Vecchio Blanco, Annalaura Torella, Teresa Giugliano, Michele Iacomino, Marcella Zollino, Giovanni Neri, Vincenzo Nigro

**Affiliations:** 1 Dipartimento di Patologia Generale, Seconda Università degli Studi di Napoli, Napoli, Italy; 2 Telethon Institute of Genetics and Medicine (TIGEM), Napoli, Italy; 3 Istituto di Genetica Medica, Università Cattolica Sacro Cuore, Policlinico A. Gemelli, Roma, Italy; Tor Vergata University of Rome, Italy

## Abstract

Critical functional properties are embedded in the non-coding portion of the human genome. Recent successful studies have shown that variations in distant-acting gene enhancer sequences can contribute to disease. In fact, various disorders, such as thalassaemias, preaxial polydactyly or susceptibility to Hirschsprung’s disease, may be the result of rearrangements of enhancer elements. We have analyzed the distribution of enhancer loci in the genome and compared their localization to that of previously described copy-number variations (CNVs). These data suggest a negative selection of copy number variable enhancers. To identify CNVs covering enhancer elements, we have developed a simple and cost-effective test. Here we describe the gene selection, design strategy and experimental validation of a customized oligonucleotide Array-Based Comparative Genomic Hybridization (aCGH), designated *Enhancer Chip*. It has been designed to investigate CNVs, allowing the analysis of all the genome with a 300 Kb resolution and specific disease regions (telomeres, centromeres and selected disease loci) at a tenfold higher resolution. Moreover, this is the first aCGH able to test over 1,250 enhancers, in order to investigate their potential pathogenic role. Validation experiments have demonstrated that *Enhancer Chip* efficiently detects duplications and deletions covering enhancer loci, demonstrating that it is a powerful instrument to detect and characterize copy number variable enhancers.

## Introduction

Recently, researchers have been focusing their efforts on the study of the non coding part of the human DNA and, in particular, on its predicted role in the regulation of gene expression [1]. In particular, comparative sequence analysis has proved to be a valuable instrument to identify regulatory elements that have been highly conserved throughout evolution [2], many of these being noncoding sequences shown to act as enhancers in experimental models [3], [4].

A database of human and mouse noncoding fragments with a gene enhancer activity has been developed [4]. VISTA Enhancer Browser is a public resource to provide access to conserved sequence elements tested for enhancer activity [5]. The database contains human candidate regions identified either by their conservation between human and non-mammalian vertebrates across long (chicken and frog) or extremely long (pufferfish and zebrafish) evolutionary distances or by their unusually high conservation among mammals, such as ‘ultra’-conservation (100% identity for at least 200 bp between human, mouse and rat) [5], [6]. Moreover, putative enhancers have been assayed for their capacity to drive reporter gene expression in a transgenic mouse model: positive enhancers are elements that drive report gene expression at mouse embryonic day 11.5 (E11.5); negative enhancers are not functional at E11.5, even though they could act as enhancers at different time points or in different physiological conditions, or their activity could depend on the presence of additional *cis*-regulatory elements.

Chromosomal rearrangements or deletions may lead to a disturbance of long-range control and, as a consequence, to pathological conditions [7]. Up to now, several alterations in enhancer structure or DNA sequence have been found to be causative of human diseases. For example, thalassaemias may be the result of deletions or rearrangements of β-globin gene (*HBB*) enhancers [8], sonic hedgehog (*SHH*) limb-enhancer point mutations can cause preaxial polydactyly [9] and a susceptibility to Hirschsprung’s disease has been associated with a *RET* proto-oncogene enhancer variant [10]. Moreover, a large number of disease susceptibility regions overlapping non-coding intervals has been mapped in genome wide association studies (GWAS) [11].

While impressive results have been obtained in the discovery and mapping of tissue specific enhancers [12], the analysis of CNVs covering these elements and their correlation with the phenotype has been hampered by the lack of a method able to detect them.

Recently, Array-Based Comparative Genomic Hybridization (aCGH) has been found to be able to detect causative alterations in patients with unexplained developmental delay/intellectual disability (DD/ID), autism spectrum disorders (ASD), and multiple congenital anomalies (MCA) in between 11% and 15% of examined cases [13]. Copy number variations in regions not investigated so far could be responsible for other undiagnosed cases.

Moreover, enhancers have been demonstrated to be located near genes active during development [11], suggesting their involvement in the regulation of these, often disease related, genes.

CNVs encompassing enhancer noncoding sequences could in this way affect target gene expression, causing human disorders.

To characterize CNVs overlapping VISTA enhancer loci, we have compared the coordinates of human VISTA enhancer loci with CNVs deposited to the Database of Genomic Variants (DGV) and Indels (small insertions and deletions of 100 bp-1 kb length) and with two highly polymorphic sets of deleted and duplicated regions. We have shown that highly polymorphic CNVs are under negative selection at VISTA enhancer loci, suggesting that copy number variable enhancers could represent functional variants. Array-CGH represents a reasonable, cost-effective instrument to investigate multiple DNA regions. To confirm the functional relevance of enhancers and to verify whether dysmorphic features or mental retardation could be associated with rare or private duplications and deletions in these elements, we have designed the *Enhancer Chip* custom array that is described below.

**Table 1:** VISTA enhancer loci localized in polymorphic CNV regions.

Vista enhancers localized in Copy Number Polymorphism (CNP) regions					
Enhancer ID	Enhancer position	Enhancer Bracketing Genes	Enhancer Expression	CNP position	CNP ID
hs98	chr16∶22684122–22685282	CDR2-HS3ST2	Negative	chr16∶22557932–22704521	2157
hs628	chr9∶159657–160780	APOA1	Positive	chr9∶149481–274606	11533
hs1108	chr9∶128945054–128946417	PBX3-FAM125B	Negative	chr9∶128944285–128954309	1512
**Vista enhancers localized in “polymorphic-DC” regions**					
**Enhancer ID**	**Enhancer position**	**Enhancer Bracketing Genes**	**Enhancer Expression**	**CNV position**	**CNV ID**
hs7	chr16∶79026563–79028162	WWOX (intragenic)	Negative	chr16∶79026434–79039202	CNVR6795.1
hs98	chr16∶22684122–22685282	CDR2-HS3ST2	Negative	chr16∶22551908–22712190	CNVR6669.1
hs445	chr1∶83878319–83879217	LPHN2-FLJ23033	Negative	chr1∶83598248–83955219	CNVR230.1
hs628	chr9∶159657–160780	APOA1	Positive	chr9∶48710–209354	CNVR4135.1
hs1339	chr9∶92292484–92293889	GADD45G-DIRAS2	Positive	chr9∶91963403–92343382	CNVR4393.2

## Methods

### Bionformatic Analysis

Genomic coordinates of 1,275 human enhancer loci, 67,419 CNVs and 34,186 Indels were downloaded from Vista enhancer database (www.enhancer.lbl.gov/) [5] and from Database of Genomic Variants (http://projects.tcag.ca/variation) [14].

The coordinates of Genomic microduplication and microdeletion syndromes were downloaded from DECIPHER database v5.1 (http://decipher.sanger.ac.uk) [15]. The coordinates of 1,319 CNVs described as Copy Number Polymorphism (CNP) and 5,037 CNVs described as “polymorphic-DC” were extracted from published supplementary materials [16], [17]. Bioinformatic data considered in this work are summarized in Table S1.

The number of enhancer loci and fraction of genome covered by CNV regions were calculated using “feature coverage” and “base coverage” tools available on the Galaxy, web portal for large-scale interactive data analyses [18].

**Figure 1:**
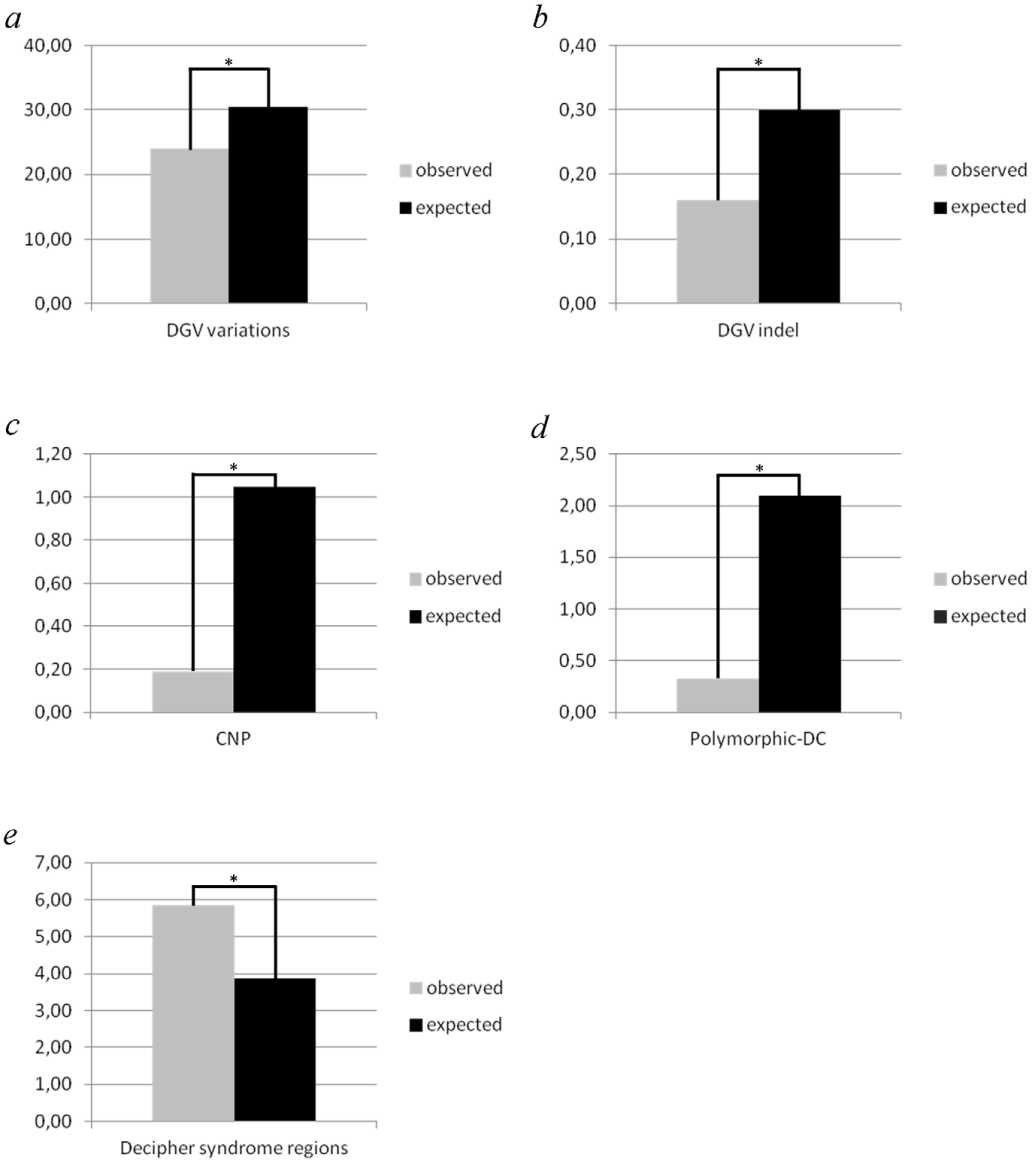
Comparison of observed and expected number (fraction) of VISTA enhancer loci located in different CNV regions. Bar graphs show the fractions of VISTA enhancer loci (observed numbers, grey bars) and the genome (expected numbers, black bars) covered by “DGV-deposited” CNV regions (**a**), “DGV-deposited” Indels (**b**), by two sets of polymorphic CNVs (**c** and **d**) and by chromosomal regions implicated in microdeletion/microduplication syndromes (Decipher syndrome regions, (**e**). All expected values were estimated based on the fraction of the genome covered by CNVs. * p-value = 2.20E−16 as calculated on absolute numbers.

### Array CGH Design

*Enhancer Chip* design was developed using the Agilent platform and the SurePrint G3 8×60K format.

Probe selection was performed using the web-based Agilent eArray database version 5.0 (https://earray.chem.agilent.com/earray/; Agilent Technologies, USA) and searching for 24.3 million computationally validated CGH oligonucleotide probe database.

Biological features were randomly distributed on the microarray and the routinely used Human CGH 1K Agilent Normalization Probe Group (1,262 features) and the Human CGH 1K Agilent Replicate Probe Group (5,000 features) were also included into design.

**Table 2:** VISTA enhancer loci overlapped by “DGV” Indels.

Enhancer ID	Enhancer position	Enhancer Bracketing Genes	Enhancer Expression	Indel position	Indel ID
hs205	chr2∶66297527–66299214	Meis1-Spred2	Positive	chr2∶66296800–66297653	Variation_115355
hs571	chr13∶112793153–112794130	Sox1-1700094C09Rik	Negative	chr13∶112793819–112794660	Variation_61360
hs808	chr18∶73570346–73571156	Zfp516-Tshz1	Negative	chr18∶73569805–73570587	Variation_61640
				chr18∶73569967–73570384	Variation_41944
hs809	chr1∶87795192–87796737	Lmo4-Hs2st1	Positive	chr1∶87796383–87797017	Variation_69038
hs855	chr11∶31989173–31990022	Rcn1-Pax6os1	Positive	chr11∶31989283–31989466	Variation_60040
hs1387	chr3∶63672828–63674786	Sntn-Thoc7	Positive	chr3∶63673334–63673795	Variation_51148
hs1592	chr20∶39461549–39463625	Mafb-Top1	Negative	chr20∶39462401–39462826	Variation_79198
				chr20∶39462851–39463076	Variation_90558
				chr20∶39462851–39463026	Variation_79197
				chr20∶39462951–39463626	Variation_79196
hs1675	chr17∶27994702–27996874	Ssh2(intragenic)	Positive	chr17∶27995436–27995598	Variation_60230

### Loci Selection

*Enhancer Chip* array was designed to provide redundancy with high sensitivity and specificity for detection of clinically significant unbalanced chromosomal abnormalities, while minimizing detection of non pathogenic CNVs or CNVs of uncertain significance. To this aim, we selected 322 diseases related to development delay or congenital physical anomalies, listed in Table S2. Moreover to further characterize CNVs overlapping VISTA enhancer loci and verify if physical anomalies or mental retardation could be associated to aberrations in these elements, we also selected 1,276 putative enhancers contained in VISTA enhancer database in July 2010, excluding those (5 enhancers) for which no Agilent probes were available.

### Array Design Strategy

For *Enhancer Chip* we designed 25,000 probes to cover all the genome (backbone) with an average spacing of 100 Kb and a resolution of 300 Kb (Figure S1 *a*). We also added 18,000 probes to cover regions of interest (telomeres, centromeres, and selected diseases loci) with an average resolution of about 40 Kb (Figure S1 *b* and *c*). This design fulfills these suggestions. Moreover, 7,790 probes were designed to investigate VISTA enhancer loci. The number of probes on each element depends from enhancer length, with at least 3 probes for each enhancer and an average spacing of 238 nt (Figure S1 *d*). The residual free space on the array (2,853 features; approximately 4.8%) was randomly filled with probes from the commercially available Agilent Human Genome 44K array CGH.

**Table 3:** Pathogenic CNV detected.

Sample	Provided by	Sex	Results	
			Agilent 4×44K (hg18)	GenomicEnhancerEnhanced (hg19)
1	Università Cattolica	F	dup 16p13.3 (3471479–4598878)*	dup 16p13.3 (3344135–4861445)
2	Università Cattolica	F	del 9q34.3 (137679200–140118015)*	del 9q34.3 (139793877–140884520)
3	Università Cattolica	F	del 17q21.3 (41073486–41566599)	del 17q21.3 (43797475–44138714)
4	Università Cattolica	F	del 11p15.5 (2246596–3110029)	del 11p15.5 (2289821–3140742)
5	Università Cattolica	F	del 13q12.12 (22464962–23788143)	del 13q12.12 (23566762–24890293)
6	Università Cattolica	F	del 9q34.3 (139407449–139633014)	del 9q34.3 (140341714–140527403)
7	Università Cattolica	F	del 6p21.32 (33396200–33696513)	del 6p21.32 (33287198–33616282)
8	Università Cattolica	F	del 15q11.2-q13.1 (20335887–26198996)	del 15q11.2-q13.1 (22885438–28387114)
9	Università Cattolica	M	dup Xq28 (149674897–154213569)	dup Xq28 (149924039–154709242)
10	Università Cattolica	M	del 1q44 (240135000–241608000)*	del 1q44 (243827851–245269686)
11	Università Cattolica	M	del 1q21.1 (144124745–145031426)	del 1q21.1 (145439778–145740798)
12	Università Cattolica	M	del 12q24.33 (131247007–132278059)	del 12q24.33 (132704696–133779599)
13	Università Cattolica	M	del 17q12 (31925709–33242217)	del 17q12 (35149154–36214168)
14	Università Cattolica	M	del 2q37,3 (240561565–242690037)	del 2q37,3 (240849992–242710613)
			dupX p22.33 - q2.8 (2782031–154494649)	dupX p22.33 - q2.8 (604588–154854819)
15	Università Cattolica	M	del 11p15.5 (2628431–2826767)**	del 11p15.5 (2671656–2870333)
16 NC	Università Cattolica	M	No pathogenic CNV detected *Partial* *ZEB2 deletion detected by MLPA*	No pathogenic CNV detected
17	Università Cattolica	F	No pathogenic CNV detected	No pathogenic CNV detected
18	Università Cattolica	F	No pathogenic CNV detected	No pathogenic CNV detected
19	Università Cattolica	M	No pathogenic CNV detected	No pathogenic CNV detected
20	Università Cattolica	M	del 13q33.1 (101213139–101288931)**	del 13q33.1 (102438335–102471438)
21	Università Cattolica	F	No pathogenic CNV detected	No pathogenic CNV detected
22	Università Cattolica	M	del 17p13.1 (7232718–7554899)	del 17p13.1 (7301795–7627091)
23	Università Cattolica	M	del 9p23 (14077982–14260484)	del 9p23 (14062880–14179942)
24	Università Cattolica	M	del 5q14.3 (89535781–90496532)	del 5q14.3 (88669375–90523605)
25	Università Cattolica	F	dup 1q44 (245107055–246198035)	dup 1q44 (247040233–247401795)
26	Università Cattolica	F	dup Xp11.3 (43456030–43979584)	dup Xp11.3 (43540470–44035710)
27 NC	Università Cattolica	M	No pathogenic CNV detected	No pathogenic CNV detected
28 NC	Università Cattolica	F	No pathogenic CNV detected	No pathogenic CNV detected
29	Università Cattolica	F	dup 14q32.12 (91361784–92145898)	dup 14q32.12 (92291831–93076286)
30	Università Cattolica	M	del 17q24.2 (62614998–63421974)	del 17q24.2 (65239676–65996750)
31	Università Cattolica	M	del 14q23.3 (66171991–66505746)	del 14q23.3 (67094610–67436135)
32	Università Cattolica	M	dup 22q11.1-q11.21 (14433473–16951255)	dup 22q11.1-q11.21 (16053273–18610161)
33	Università Cattolica	F	No pathogenic CNV detected	No pathogenic CNV detected
34	Università Cattolica	F	No pathogenic CNV detected	No pathogenic CNV detected
35	Università Cattolica	F	No pathogenic CNV detected	No pathogenic CNV detected
36 NC	Università Cattolica	M	del 3q24 (150191621–150351168) *Deletion <0.3 Kb*	No pathogenic CNV detected
37	Università Cattolica	M	del Xp21.1 (31745716–31990165)	del Xp21.1 (31745716–31990165)
38	Università Cattolica	F	No pathogenic CNV detected	No pathogenic CNV detected
39	Università Cattolica	F	del 14q23.3 (66299338–66460789)	del 14q23.3 (67204662–67351828)
40	Università Cattolica	F	No pathogenic CNV detected	No pathogenic CNV detected
118	Seconda Univesità	M	Not analyzed *NF1 deletion detected by MLPA*	del 17q11.2 (29024152–30367355)
IM	Seconda Univesità	F	Not analyzed *Uncharacterized 6p duplication by G-banding*	dup 6p21.31 - p12.3 (35489309–49273592)
IF	Seconda Univesità	F	Not analyzed *Uncharacterized 6p duplication by G-banding*	dup 6p21.31 - p12.3 (35489309–49273592)
NB	Seconda Univesità	F	Not analyzed	No pathogenic CNV detected
GF	Seconda Univesità	F	Not analyzed	No pathogenic CNV detected
RS	Seconda Univesità	F	Not analyzed *Mosaic X-deletion by G-banding*	del X
X926	Seconda Univesità	M	Not analyzed	No pathogenic CNV detected

* BAC array 1 Mb.

** Agilent 244K.

NC = negative control.

### Reference DNA Samples

Anonymous blood samples were collected from healthy, unrelated Italian individuals. All subjects studied entered the diagnostic centers of Naples or Rome and signed an appropriate consent form for genetic testing as well as forms related to privacy of data. Approval for the study was obtained by the Seconda Università di Napoli Ethics Committee (prot. 862/08). Genomic DNA was extracted using standard procedures.

After amelogenin-based sex confirmation by PCR, concentration and purity of DNA samples were assessed using Nanodrop ND 1000 (Thermo Scientific Inc., USA) by evaluating the A260/A280 and A260/A230 ratios to exclude contaminating proteins or other organic compounds. After dilution to a final concentration of 100 ng/µL, six sex-matched DNA samples were pooled together and used as male or female reference DNA samples in array CGH experiments.

### DNA Samples

For validation experiments, we utilized DNA samples from patients in which genomic deletions or duplications were previously detected with alternative diagnostic methods. In addition, we also utilized DNA samples from patients with development delay and/or congenital anomalies but without a molecular diagnosis. Further DNA samples from healthy individuals were also analyzed.

In particular, forty DNA samples were collected at Università Cattolica del Sacro Cuore (Rome) and seven at the Seconda Università di Napoli (Naples).

For all DNA samples, sex was confirmed by amelogenin-based PCR assay. Concentration and purity of each DNA sample were assessed using Nanodrop ND 1000 (Thermo Scientific Inc., USA). After dilution to a final concentration of 100 ng/µL, all DNA samples were blindly tested in array CGH experiments.

**Table 4:** VISTA enhancer loci deleted in our patients.

Enhancer ID	Enhancer position	Sample	Chromosome region*	Enhancer Bracketing Genes	Enhancer Expression	Expression Pattern
hs607	chr12∶16,610,045–16,611,936	27	chr12∶16606713–16665783	MGST1-LMO3	Positive	hindbrain (rhombencephalon), neural tube
hs676	chr6∶97,544,611–97,545,759	25	chr6∶97375836–97550891	KIAA1900 (intragenic)	Positive	branchial arch, ear, forebrain, hindbrain
hs775	chr18∶77,010,009–77,010,795	01	chr18∶77008844–77024003	CDR2-HS3ST2	Positive	forebrain

* deleted regions as detected by sequencing.

### Array CGH Hybridization and Analysis

Labeling and hybridization were performed according to the manufacturer’s specifications (Agilent Oligonucleotide Array-Based CGH for Genomic DNA Analysis protocol, version 6.1; Agilent Technologies, USA). Scanned array images were analyzed with Feature Extraction software (version 10.5.1.1; Agilent Technologies, USA). Graphical overview and analysis of data were obtained using DNA Analytics as part of Agilent Genomic Workbench software (version 5.0; Agilent Technologies, USA), evaluating the quality of each test with the quality control (QC) metrics generated with DNA Analytics software. For identifying duplications and deletions we used the standard set-up of the Aberration Detection Method 2 (ADM-2) algorithm for the data that passed QC metrics testing. The threshold applied to the algorithm was empirically chosen for each test. An aberration filter was set to select aberrant regions with at least 3 targets showing the same direction in copy-number change and to exclude aberrant regions if the average log2 ratio within the region was less than the value of Derivative Log Ratio spread (DLRSpread). Variants not known to be pathogenic were compared with the Database of Genomic Variants (http://projects.tcag.ca/variation/) and with the Decipher database (http://decipher.sanger.ac.uk/) to facilitate interpretation.

**Figure 2:**
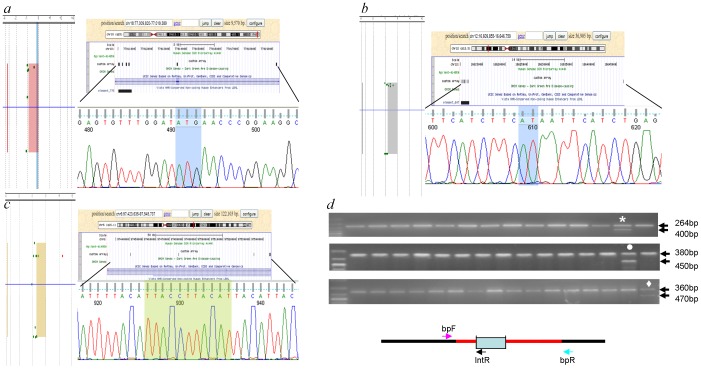
New CNVs overlapping VISTA enhancers. aCGH results for sample 1 (**a**), 25 (**b**) and 27 (**c**) showing deletions overlapping enhancers hs775, hs676 and hs607, respectively. (**d**) Genotyping assay for deletions in enhancers hs775 (upper panel), hs676 (middle panel) and hs607 (lower panel): triplex PCR with a forward primer (bpF) located 5' to the breakpoint, a reverse primer within the deleted region (in red) and a second one 3′ the distal breakpoint (box represents the enhancer elements). Bands of 264 bp, 380 bp and 360 bp, respectively, correspond to bpF-IntR amplicons, the lower bands represent bpF-bpR amplicons. Only sample 1 (* in upper panel), 25 (• in middle panel) and 27 (♦ in lower panel) show, respectively, the double bands, all the other analyzed samples show a normal pattern.

All validation experiments were carried out by comparison with male or female reference DNA samples obtained by pooling of six sex-matched genomic DNA samples from healthy and unrelated individuals of the same ethnic origin [19].

### Real Time PCR, Long- range PCR and DNA Sequencing

Real Time PCR reactions were performed using ***Bio-Rad iQ SYBR*** Green Supermix with 1 ng of DNA, according to the manufacturer’s specifications. The PCR conditions were the following: 96°C×1 min; 45 cycles of 96°C×30 s, 62°C×30 s and 68°C×30 s, with 72°C×12 min as final step.

Long-range PCR reactions were performed using 100 ng DNA, 0.5 µM of each primer, 400 µM of each dNTP, and 1.5 units TaKaRa LA Taq in 1X LA PCR Buffer II (TaKaRa Bio, Inc., Japan). The PCR conditions were the following: 96°C×1 min; 30 cycles of 96°C×30 s, 62°C×1 min and 68°C×4 min plus 5 s/cycle, with 72°C×12 min as final step.

PCR products were double-strand sequenced using BigDye Terminator sequencing chemistry (Life Technologies, USA) and analyzed on an ABI 3130×L automatic DNA sequencer (Applied Biosystems, USA).

## Results

### Relationship between VISTA Enhancer Loci and CNV Regions

To verify whether the characterized CNVs overlap the VISTA enhancers, we compared the positions of their loci with three sets of CNVs: ‘DGV-deposited’ (N = 67,419; 30.37% genome coverage) [14], “polymorphic-CNP” (N = 1,319; 1.06% genome coverage) [16] and “polymorphic-DC” (N = 5,037; 2.3% genome coverage) [17] CNVs. “DGV-deposited” CNVs include all 67,419 CNVs deposited in the Database of Genomic Variants (DGV update Nov 02, 2010– variation.hg18.v10.nov.2010.txt, file available at http://projects.tcag.ca/variation). “Polymorphic-CNP” [16] and “polymorphic-DC” [17] are two sets of highly polymorphic CNVs (minor allele frequency >0.01) validated by high-quality genotyping in two CNV-discovery studies using high-density arrays. In both of these studies, precise breakpoints and unambiguous copy numbers were determined for each analyzed sample.

We identified 326 VISTA enhancer loci localized in “DGV-deposited” CNVs (Table S3) and 3 in CNP and 5 in “polymorphic-DC” CNV regions (Table 1).

Next, we determined whether the overlap of the CNVs and enhancer loci was random (null hypothesis) or whether the CNVs were underrepresented at these loci (alternative hypothesis). To test these hypotheses, we compared fractions of the enhancer loci and fractions of the genome covered by the differentially defined CNV regions. Figure 1 (*b* and *c*) shows that the fractions of the enhancer loci (0.19% and 0.33%) covered by the two sets of “polymorphic” CNVs are at least five times lower than the fractions of the covered genome (1.05% and 2.10%). Also the 34,186 small Indels deposited in the DGV (with a genomic coverage of 0.3%) overlap the VISTA enhancer loci two-fold lower than expected (0.16% corresponding to 8 enhancers, see Figure 1*b* and Table 2 for a complete list).

These data demonstrate a negative selection of highly polymorphic CNVs and of small Indels at enhancer loci.

The CNV purification effect is less strong if one compares the fraction of the enhancers (24%) covered by the “DGV-deposited” CNVs with the fraction of the genome covered by those CNVs (30%)(Figure 1*a*). As already discussed in recently published papers [16], [20], the low purifying effect observed for the “DGV-deposited” CNVs suggests that some of these CNVs are very rare or private or could be false positive artifacts.

Finally, not only common CNVs but also CNVs implicated in specific diseases can affect enhancer loci and thus can play an important role in pathogenesis. We have identified 77 enhancers (Table S4) located in chromosomal regions implicated in microdeletion/microduplication syndromes (DECYPHER v5.0) [15]. The role of enhancer CNVs in the pathogenesis of these conditions has never been investigated.

### Validation Strategy of the *Enhancer Chip*

For the *Enhancer Chip* validation, we analyzed 47 blind DNA samples (Table 3). These included 31 samples from patients in whom genomic imbalances had previously been detected by aCGH (27/31), G-banding (3/31) or MLPA (1/31) and 12 from patients with a clinical diagnosis of development delay or congenital anomalies (with the exception of GF and X926 who each had a prevalent muscular phenotype). Two samples (16 and 36) from affected individuals with deleterious mutations undetectable with *Enhancer Chip* and two (27 and 28) from healthy individuals were also included as negative controls.

The *Enhancer Chip* detected no clinically relevantgenomic imbalances in any of the 4 negative controls and confirmed the molecular diagnosis in 31 out of 31 samples, that had previously been analyzed (Table 3). Sample 15 presented a heterozygous intragenic deletion of the *KCNQ1*gene (MIM 607542) and sample 20 showed a heterozygous intragenic deletion of the *FGF14* gene (MIM 601515). These variations, undetectable by Agilent 44K array, had been identified by using a 244k Agilent array and confirmed by using the *Enhancer Chip*. We also revealed a NF1 deletion (118) detected by MLPA and a 6p duplication (IM and IF) detected by G-banding. In these cases our array proved to be a valuable tool to define breakpoint boundaries and the extension of the aberrations (data not shown), demonstrating that it is a good alternative to commercial all-genome platforms.

In 2 out of 12 patients (17–21) with only a clinical diagnosis, the *Enhancer Chip* array was able to detect new small copy-number changes, classified as variants of uncertain clinical significance (Table S5).

Finally, the *Enhancer Chip* detected 3 deletions (Table 4) that overlap the VISTA enhancer loci (hs775, hs676, hs607) in 3 different samples (1, 25 and 27 respectively).

In patient 1 (Figure 2*a*) the result was confirmed by long-range PCR and direct sequencing, showing an insertion of 3 nucleotides at breakpoint boundaries.

In samples 25 and 27 (Figures 2*b*–*c*) the extension of these deletions was confirmed and better defined by Real-Time PCR. Finally, the breakpoints were finely mapped by direct sequencing, demonstrating a 11-bp long and 2-bp long insertion in samples 25 and 27, respectively.

Interestingly, all three enhancers are active at stage E11.5. In particular, hs775 drives the reporter gene expression in the forebrain of transgenic mice, hs676 in the branchial arch, ear, forebrain and hindbrain, and hs607 in the hindbrain (rhombencephalon) and neural tube (Table 4). None of these rearrangements have been described and there are no CNVs deposited in the DGV overlapping these three enhancers.

To genotype these 3 new deletions, we developed a triplex PCR assay (Figure 2*d*). The forward primer was located 5′ to the breakpoint, the reverse primer for detecting the wild type allele within the deletion sequence and the reverse primer for detecting the deletion 3′ to the breakpoint. This enabled us to specifically amplify both the wild type and the deletion alleles, even in heterozygous samples. We carried out this analysis on 300 samples to genotype the deletions detected in samples 1, 25 and 27 and we confirmed these to be rare or private losses.

## Discussion

“Polymorphic” CNVs show some purifying effects at VISTA enhancer loci, as already seen for miRNA genes [21], which are equally underrepresented in polymorphic copy number variable regions. As indicated in Table 1, only a small fraction of CNV-enhancers has been identified so far. The enhancers are not only conserved elements across evolution but also relatively stable among humans.

Although it is very difficult to predict how many highly polymorphic CNV-enhancers are present in the human genome, they are potential functional variants and could represent candidate loci, especially if located in regions implicated in diseases by linkage or association studies. A previous study [22] has shown that a deletion of several ultraconserved non-coding sequences in mice may not result in obvious phenotypes, demonstrating that even an extreme evolutionary constraint does not necessarily indicate that a non-coding sequence is required for viability. The general opinion is that non-coding conserved sequences are essential and that their deletion may result in severe phenotypes. This lack of an obvious effect could be due to several considerations, similar to those that could explain the absence of a phenotype upon deletion of highly conserved protein-coding genes: minor phenotypes not detected; a functional redundancy with other genes or enhancers; or reductions in fitness that only become apparent over multiple generations or are not easily detected in a controlled laboratory environment [11]. However, contrary to this finding, the number of recorded cases of non-coding mutations linked to human diseases has been growing rapidly. Several chromosomal alterations demonstrate a link between malformations and regulatory mutations. Aniridia and related eye anomalies may arise from chromosomal rearrangements that disrupt the region downstream of the *PAX6* transcription unit [23]. A number of long-range regulatory disruptions are associated with genes of the forkhead/winged helix group of transcription factors, such as FOXC1, FOXC2 and FOXL2, causing ocular malformations [24]. Chromosomal rearrangements can remove one or more cis-regulatory elements of the *SOX9* gene, leading to campomelic dysplasia [25]. Holoprosencephaly has also been associated with long-range regulator mutations leading to a haploinsufficiency of SIX3 or SHH proteins [26].

The lack of precise data on CNV-enhancers, their polymorphisms and their putative pathogenic role is mostly due to the absence of appropriate methods for their identification and characterization in a large number of samples. A simple and inexpensive method that enables an accurate characterization of several CNVs of interest has never been proposed up to now, hampering the analyses of CNVs and their correlation with the phenotype.

In order to characterize all CNV-enhancers and eventually identify cryptic disease-associated deletions or duplications, we have developed our *Enhancer Chip*, a straightforward and cost-effective assay with research purposes. A custom-designed array represents an important diagnostic instrument [27], as well as a powerful technique to identify novel disease genes or to characterize relatively unknown elements. Validation experiments, here described, demonstrate its sensitivity and specificity, confirming all the results generated by other methods. Moreover, thanks to probes on the VISTA enhancer loci, our custom array is an innovative tool, the first one to investigate enhancer elements. Clearly, we have designed this array not as a tool for molecular diagnosis, but to discover new CNVs covering enhancers and potentially causing disease phenotypes. As described, the *Enhancer Chip* has allowed the detection of three new and supposedly rare deletions covering enhancers. These three enhancers have been demonstrated to be active during embryonic development even if nothing is known about their gene targets. Our samples show heterozygous deletions on these elements. It would be interesting to evaluate the expression levels of their putative targets, once identified. Probably none of the three alterations is directly responsible for the observed phenotypes in these patients. Further studies on a large population could help to identify the phenotypic effects of copy variable enhancers.

Other recent papers have demonstrated the utility of an exon-targeted oligonucleotide array (i.e., aCGH using an array with probes concentrated disproportionately in the exons) to detect intragenic copy-number changes in patients with various clinical phenotypes [28], [29]. An exon-targeted design improves the resolution of aCGH to the level of the exon while excluding much of the noise inherent in other strategies. Our subsets of experimentally validated probes covering the VISTA enhancer loci could be used in any dedicated exon-targeted array design.

Although we have focused on distant-acting enhancers, there are other categories of functional elements in the non-coding portion of the genome (for example insulators, negative regulators, promoters and non-coding RNAs), which are also crucial targets for a large scale study of regulatory elements in the human genome. To identify deletions or duplications in all these elements, we are going to develop a new customized CGH-array, to investigate these regulatory regions.

## Supporting Information

Figure S1 Probe selection strategy for *Enhancer Chip* design.
(DOC)

Table S1 Bioinformatic data analysed.
(DOC)

Table S2 Disease loci investigated by *Enhancer Chip* array.
(DOC)

Table S3 VISTA enhancer loci localized in *DVG* variations.
(DOC)

Table S4 VISTA enhancer loci localized in Decipher Syndromes Regions.
(DOC)

Table S5 CNVs of uncertain significance detected by *Enhancer Chip.*
(DOC)

## References

[1] Klopocki E, Mundlos S (2011) Copy-number variations, noncoding sequences, and human phenotypes. Annu Rev Genomics Hum Genet 12: 53–72. 21756107 10.1146/annurev-genom-082410-101404

[2] Narlikar L, Ovcharenko I (2009) Identifying regulatory elements in eukaryotic genomes. Brief Funct Genomic Proteomic 8: 215–230. 19498043 10.1093/bfgp/elp014 PMC2764519

[3] Woolfe A, Goodson M, Goode DK, Snell P, McEwen GK, et al (2005) Highly conserved non-coding sequences are associated with vertebrate development. PLoS Biol 3: e7. 15630479 10.1371/journal.pbio.0030007 PMC526512

[4] Pennacchio LA, Ahituv N, Moses AM, Prabhakar S, Nobrega MA, et al (2006) In vivo enhancer analysis of human conserved non-coding sequences. Nature 444: 499–502. 17086198 10.1038/nature05295

[5] Visel A, Minovitsky S, Dubchak I, Pennacchio LA (2007) VISTA Enhancer Browser–a database of tissue-specific human enhancers. Nucleic Acids Res 35: D88–92. 17130149 10.1093/nar/gkl822 PMC1716724

[6] Bejerano G, Pheasant M, Makunin I, Stephen S, Kent WJ, et al (2004) Ultraconserved elements in the human genome. Science 304: 1321–1325. 15131266 10.1126/science.1098119

[7] Kleinjan DA, van Heyningen V (2005) Long-range control of gene expression: emerging mechanisms and disruption in disease. Am J Hum Genet 76: 8–32. 15549674 10.1086/426833 PMC1196435

[8] Kioussis D, Vanin E, deLange T, Flavell RA, Grosveld FG (1983) Beta-globin gene inactivation by DNA translocation in gamma beta-thalassaemia. Nature 306: 662–666. 6318113 10.1038/306662a0

[9] Lettice LA, Heaney SJ, Purdie LA, Li L, de Beer P, et al (2003) A long-range Shh enhancer regulates expression in the developing limb and fin and is associated with preaxial polydactyly. Hum Mol Genet 12: 1725–1735. 12837695 10.1093/hmg/ddg180

[10] Grice EA, Rochelle ES, Green ED, Chakravarti A, McCallion AS (2005) Evaluation of the RET regulatory landscape reveals the biological relevance of a HSCR-implicated enhancer. Hum Mol Genet 14: 3837–3845. 16269442 10.1093/hmg/ddi408

[11] Visel A, Rubin EM, Pennacchio LA (2009) Genomic views of distant-acting enhancers. Nature 461: 199–205. 19741700 10.1038/nature08451 PMC2923221

[12] May D, Blow MJ, Kaplan T, McCulley DJ, Jensen BC, et al (2011) Large-scale discovery of enhancers from human heart tissue. Nat Genet 44: 89–93. 22138689 10.1038/ng.1006 PMC3246570

[13] Miller DT, Adam MP, Aradhya S, Biesecker LG, Brothman AR, et al (2010) Consensus statement: chromosomal microarray is a first-tier clinical diagnostic test for individuals with developmental disabilities or congenital anomalies. Am J Hum Genet 86: 749–764. 20466091 10.1016/j.ajhg.2010.04.006 PMC2869000

[14] Iafrate AJ, Feuk L, Rivera MN, Listewnik ML, Donahoe PK, et al (2004) Detection of large-scale variation in the human genome. Nat Genet 36: 949–951. 15286789 10.1038/ng1416

[15] Firth HV, Richards SM, Bevan AP, Clayton S, Corpas M, et al (2009) DECIPHER: Database of Chromosomal Imbalance and Phenotype in Humans Using Ensembl Resources. Am J Hum Genet 84: 524–533. 19344873 10.1016/j.ajhg.2009.03.010 PMC2667985

[16] McCarroll SA, Kuruvilla FG, Korn JM, Cawley S, Nemesh J, et al (2008) Integrated detection and population-genetic analysis of SNPs and copy number variation. Nat Genet 40: 1166–1174. 18776908 10.1038/ng.238

[17] Conrad DF, Pinto D, Redon R, Feuk L, Gokcumen O, et al (2010) Origins and functional impact of copy number variation in the human genome. Nature 464: 704–712. 19812545 10.1038/nature08516 PMC3330748

[18] Taylor J, Schenck I, Blankenberg D, Nekrutenko A (2007) Using galaxy to perform large-scale interactive data analyses. Curr Protoc Bioinformatics Chapter 10: Unit 10 15. 10.1002/0471250953.bi1005s19 PMC3418382 18428782

[19] Yatsenko SA, Shaw CA, Ou Z, Pursley AN, Patel A, et al (2009) Microarray-based comparative genomic hybridization using sex-matched reference DNA provides greater sensitivity for detection of sex chromosome imbalances than array-comparative genomic hybridization with sex-mismatched reference DNA. J Mol Diagn 11: 226–237. 19324990 10.2353/jmoldx.2009.080064 PMC2671340

[20] Itsara A, Cooper GM, Baker C, Girirajan S, Li J, et al (2009) Population analysis of large copy number variants and hotspots of human genetic disease. Am J Hum Genet 84: 148–161. 19166990 10.1016/j.ajhg.2008.12.014 PMC2668011

[21] Marcinkowska M, Szymanski M, Krzyzosiak WJ, Kozlowski P (2011) Copy number variation of microRNA genes in the human genome. BMC Genomics 12: 183. 21486463 10.1186/1471-2164-12-183 PMC3087710

[22] Ahituv N, Zhu Y, Visel A, Holt A, Afzal V, et al (2007) Deletion of ultraconserved elements yields viable mice. PLoS Biol 5: e234. 17803355 10.1371/journal.pbio.0050234 PMC1964772

[23] Crolla JA, van Heyningen V (2002) Frequent chromosome aberrations revealed by molecular cytogenetic studies in patients with aniridia. Am J Hum Genet 71: 1138–1149. 12386836 10.1086/344396 PMC385089

[24] Lehmann OJ, Sowden JC, Carlsson P, Jordan T, Bhattacharya SS (2003) Fox’s in development and disease. Trends Genet 19: 339–344. 12801727 10.1016/S0168-9525(03)00111-2

[25] Pfeifer D, Kist R, Dewar K, Devon K, Lander ES, et al (1999) Campomelic dysplasia translocation breakpoints are scattered over 1 Mb proximal to SOX9: evidence for an extended control region. Am J Hum Genet 65: 111–124. 10364523 10.1086/302455 PMC1378081

[26] Belloni E, Muenke M, Roessler E, Traverso G, Siegel-Bartelt J, et al (1996) Identification of Sonic hedgehog as a candidate gene responsible for holoprosencephaly. Nat Genet 14: 353–356. 8896571 10.1038/ng1196-353

[27] Wong LJ, Dimmock D, Geraghty MT, Quan R, Lichter-Konecki U, et al (2008) Utility of oligonucleotide array-based comparative genomic hybridization for detection of target gene deletions. Clin Chem 54: 1141–1148. 18487280 10.1373/clinchem.2008.103721

[28] Boone PM, Bacino CA, Shaw CA, Eng PA, Hixson PM, et al (2010) Detection of clinically relevant exonic copy-number changes by array CGH. Hum Mutat 31: 1326–1342. 20848651 10.1002/humu.21360 PMC3158569

[29] Piluso G, Dionisi M, Del Vecchio Blanco F, Torella A, Aurino S, et al (2011) Motor chip: a comparative genomic hybridization microarray for copy-number mutations in 245 neuromuscular disorders. Clin Chem 57: 1584–1596. 21896784 10.1373/clinchem.2011.168898

