# Supplementary material for: Enhancer Chip: Detecting Human Copy Number Variations in Regulatory Elements
Source: PLoS One. 2012 Dec 20;7(12):e52264. doi: 10.1371/journal.pone.0052264 (PMC3527541; doi:10.1371/journal.pone.0052264)
Supplement: Figure S1 — Probe selection strategy for Enhancer Chip design. (DOC) [file pone.0052264.s001.doc]

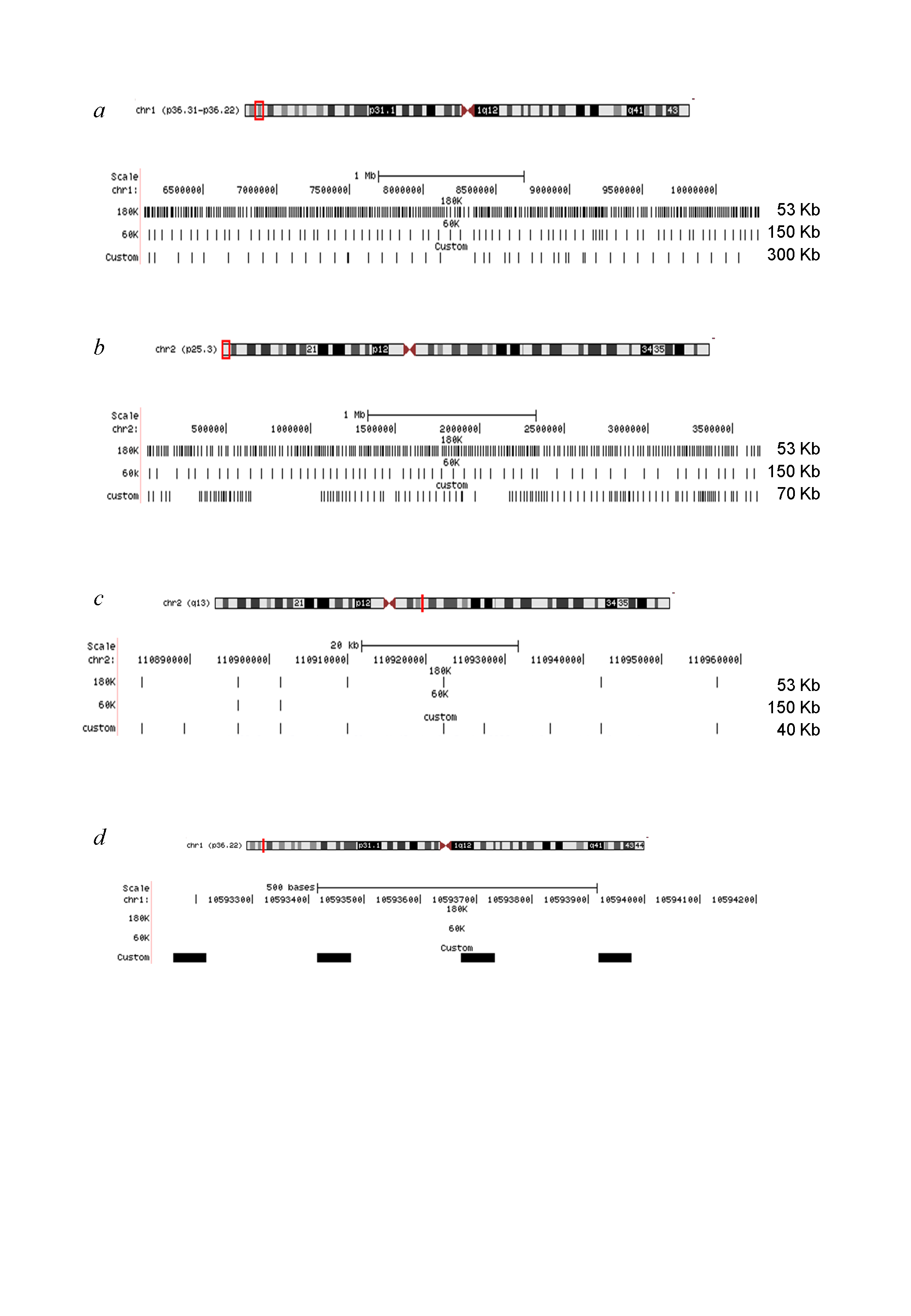


**Supplementary Figure S1 -** Probe selection strategy for *Enhancer Chip* design***.***

Schematic representation (with graphical presentation in UCSC) of probes covering genome backbone (**a**), subtelomeric regions (**b**), a mendelian disease gene (PNP, **c**) and a VISTA enhancer (**d**) for commercially available Agilent 180k, Agilent 60K and our custom array.
