# Supplementary material for: Enhancer Chip: Detecting Human Copy Number Variations in Regulatory Elements
Source: PLoS One. 2012 Dec 20;7(12):e52264. doi: 10.1371/journal.pone.0052264 (PMC3527541; doi:10.1371/journal.pone.0052264)
Supplement: Table S1 — Bioinformatic data analysed. (DOC) [file pone.0052264.s002.doc]

**Supplementary Table S1 -** Bioinformatic data analyzed

| **Target** | **Database** | **Number of features** |
| --- | --- | --- |
| Enhancer | Vista enhancer database (www.enhancer.lbl.gov/) | 1.275 |
| Copy Number Variations (CNVs) | Database of Genomic Variants (http://projects.tcag.ca/variation) | 67.419 |
| Indels | Database of Genomic Variants (http://projects.tcag.ca/variation) | 34.186 |
| Genomic microduplication and microdeletion syndromes | DECIPHER database v5.1 (http://decipher.sanger.ac.uk) | 58 |
| Polymorphic Copy Number Variations | Copy Number Polymorphism (CNP) [16] | 1.319 |
| Polymorphic Copy Number Variations | Polymorphic-DC [17] | 5.037 |
