# Supplementary material for: Enhancer Chip: Detecting Human Copy Number Variations in Regulatory Elements
Source: PLoS One. 2012 Dec 20;7(12):e52264. doi: 10.1371/journal.pone.0052264 (PMC3527541; doi:10.1371/journal.pone.0052264)
Supplement: Table S2 — Disease loci investigated by Enhancer Chip array. (DOC) [file pone.0052264.s003.doc]

**Supplementary Table S2 -** Disease loci investigated by *Enhancer Chip* array

| **Disease** | **Involved genes** | **Position** |
| --- | --- | --- |
| 1p36 Microdeletion | Multiple | 1p36 |
| 1q21.1 Microdeletion with TAR | Multiple | 1q21.1 |
| 1q21.1 Microdeletion with susceptibility to mental retardation, autism, or congenital anomalies | Multiple | 1q21.1 |
|  | ACP6 candidate |  |
|  | GJA5 candidate |  |
|  | GJA8 candidate |  |
| 1q41-q42 Microdeletion | Multiple | 1q41 |
|  | DISP1 candidate |  |
| 1q44 Microdeletion | Multiple | 1q44 |
|  | AKT3 candidate |  |
| Van der Woude | IRF6 | 1q32.2 |
| Short stature, pituitary and cerebellar defects, & small sella turcica | LHX4 | 1q25.2 |
| Stickler syndrome, type II (STL2) | COL11A1 | 1p21.1 |
| Thrombocytopenia-Absent Radius syndrome |  | 1q21.1 |
| 2p15-p16.1 Microdeletion | Multiple | 2p15-p16.1 |
| 2p21 Microdeletion, homozygous | Multiple | 2p21 |
| 2q32.2-q33 Microdeletion | Multiple | 2q33.1 |
|  | SATB2 candidate |  |
| Albright hereditary osteodystrophy-like/Brachydactyly-MR | Multiple | 2q37.3 |
| Feingold | MYCN | 2p24.3 |
| Holoprosencephaly 2 | SIX3 | 2p21 |
| Hypotonia-cystinuria | SLC3A1 | 2p21 |
|  | PREPL |  |
| Joubert 4 | NPHP1 | 2q13 |
| Mowat-Wilson * | ZFHX1B | 2q22.3 |
| Nephronophthisis 1 | NPHP1 | 2q13 |
| Severe myoclonic epilepsy of infancy (SMEI) | SCN1A | 2q24.3 |
| Synpolydactyly/Syndactyly II | HOXD gene cluster | 2q31.1 |
| Waardenburg I | PAX3 | 2q36.1 |
| Hypertension with CHD | BMPR2 | 2q33.1 |
| Holoprosencephaly 9 (HPE9) | GLI2 | 2q14.2 |
| Holoprosencephaly 6 (HPE6) |  | 2q37.1q37.3 |
| Heterotaxy 2 | CFC1 | 2q21.1 |
| Epilepsy with febrile seizure plus (GEFS+) | SCN1A | 2q24.3 |
| Cleft palate, isolated (CPI) | SATB2 | 2q32 |
| Cystinuria with mitochondrial disaese | SLC3A1 | 2p21 |
| 2q37 monosomy |  | 2q37 |
| 3q29 Microdeletion | Multiple | 3q29 |
| Blepharophimosis, ptosis epicanthus inversus (BPE) | FOXL2 | 3q22.3 |
| Dandy-Walker malformation (DWM) | ZIC1  ZIC4 | 3q24 |
| von Hippel-Lindau | VHL | 3p25.3 |
| Waardenburg IIA | MITF | 3p14.1 |
| Forebrain defects; TDGF1 | CRIPTO | 3p21.31 |
| Loeys-Dietz syndrome (LDS) | TGFBR2 | 3p24.1 |
| Marfran syndrome, Type II (MFS2) | TGFBR2 | 3p22 |
| Noonan syndrome | RAF1 | 3p25.1 |
| Split-hand/foot malformation-4 (SHFM4) | TP73L | 3q28 |
| Wolf-Hirschhorn | Multiple | 4p16.3 |
| Rieger 1 (RIEG1) | PITX2 | 4q25 |
| 5q22 Microdeletion/Gardner/Familial adenomatous polyposis with MR | APC | 5q22.2 |
| Boston-type craniosynostosis | MSX2 | 5q35.2 |
| Parietal foramina 1 | MSX2 | 5q35.2 |
| Cri-du-Chat | Multiple | 5p15.2 |
| Sotos | NSD1 | 5q35.3 |
| Autosomal Dominant Leukodystrophy | LMNB1 | 5q23.2 |
| Chronic Pancreatitis | SPINK1 | 5q32 |
| Cornelia de Lange syndrome (CDLS) | NIPBL | 5p13.2 |
| Microcephaly, congenital heart disease | NKX2-5 | 5q35.1 |
| Treacher Collins-Franceschetti syndrome | TCOF1 | 5q32 |
| Holoprosencephalia FBXW11 | FBXW11 | 5q35.1 |
| 6p25.3 Microdeletion | Multiple | 6p25.3 |
| 6q24.3 Microdeletion | Multiple | 6q24.3 |
| Cleidocranial dysplasia (CCD) | RUNX2 | 6p12.3 |
| Prader-Willi-like phenotype | SIM1 | 6q16.3 |
| Diabetes Mellitus, transient neonatal, 1 | ZAC | 6q24.2 |
| Axenfeld-Rieger syndrome | FKHL7 (FOXC1) | 6p25.3 |
| Sacral/anorectal malformation syndrome |  | 6q25.3 |
| Vascular endothelial growth factor (VEGF) | VEGF | 6p21.1 |
| 7q11.23 Microduplication | Multiple | 7q11.23 |
| Currarino | MNX1 | 7q36.3 |
| Greig cephalopolysyndactyly | GLI3 | 7p14.1 |
| Holoprosencephaly 3 | SHH | 7q36.3 |
| Infantile spasms, MAGI2-related | MAGI2 | 7q21.11 |
| Saethre-Chotzen | TWIST1 | 7p21.1 |
| Speech & language disorder 1 | FOXP2 | 7q31.1 |
| Split-hand/foot malformation 1 (SHFM1) | SHFM1 | 7q21.3 |
| Williams-Beuren | ELN | 7q11.23 |
| Osteogenesis imperfecta congenita | COL1A2 | 7q21.3 |
| Schizophrenia & epilepsy | CNTNAP2 | 7q35 |
| 8p23.1 Microdeletion | Multiple | 8p23.1 |
|  | GATA4 candidate |  |
| Branchio-oto-renal/Melnick-Fraser | EYA1 | 8q13.3 |
| CHARGE | CHD7 | 8q12.2 |
| Congenital diaphragmatic hernia 2 (CDH2) | GATA4 candidate | 8p23.1 |
| Langer-Giedion | TRPS1 | 8q23.3 |
|  | EXT1 | 8q24.11 |
| Nablus mask-like facial | Multiple | 8q21.3-q22.1 |
| Oto-facio-cervical (OFC) | EYA1 | 8q13.3 |
| Trichorhinophalangeal 1 | TRPS1 | 8q23.3 |
| 8p23p22 deletion/duplication syndrome |  | 8p22 |
| Bipolar disorder with 8q21.13 deletion | IMPA1 | 8q21.13 |
| Tetralogy of Fallot | ZFPM2/FOG2 | 8q23.1 |
| 9q22.32-q22.33 Microdeletion | Multiple (TGFBR1 candidate) | 9q22.33 |
| 9q34 Microdeletion | Multiple (EHMT1 candidate) | 9q34.3 |
| Basal cell nevus/Gorlin-Goltz | PTCH1 | 9q22.32 |
| Holoprosencephaly 7 | PTCH1 | 9q22.32 |
| Nail-patella (NPS) | LMX1B | 9q33.3 |
| Sex reversal, autosomal dominant 2 (SRA2) | Multiple (DMRT1 candidate) | 9p24.3 |
| Tuberous sclerosis 1 (TSC1) | TSC1 | 9q34.13 |
| 9p23 deletion syndrome/Trigonocephaly |  | 9p23 |
| Loeys-Dietz syndrome (LDS) | TGFBR1 | 9q22.33 |
| Sex reversal XY +/- adrenal failure | NR5A1 (SF1) | 9q33 |
| 9q subtelomeric deletion syndrome |  |  |
| 10q22.3-q23.31 Microdeletion | Multiple | 10q22.3-q23.31 |
| Bannayan-Riley-Ruvalcaba (BRRS) | PTEN | 10q23.31 |
| Cowden | PTEN | 10q23.31 |
| DiGeorge 2 | Multiple | 10p14 |
| Hypoparathyroidism, sensorineural deafness, renal disease (HDR) | GATA3 | 10p14 |
| PTEN hamartoma tumor | PTEN | 10q23.31 |
| Split-hand/foot malformation 3 (SHFM3) | FBXW4 | 10q24.32 |
| Chromosome 10q deletion syndrome |  | 10q26 |
| Hirschsprung | RET | 10q11.21 |
| Nebulette | NEBL | 10p12.31 |
| Charcot-Marie-Tooth disease type 1D | EGR2 |  |
| Aniridia II | PAX6 | 11p13 |
| Beckwith-Wiedemann, IGF2-related * | IGF2 | 11p15.5 |
| Jacobsen/11q terminal deletion disorder | Multiple | 11q23 -11qter |
| Oto-dental | FGF3 | 11q13.3 |
| Potocki-Shaffer | EXT2 ALX4 | 11p11.2 |
| WAGR | PAX6 WT1 | 11p13 |
| Wilms Tumor 1 | WT1 | 11p13 |
| Craniosynostosis with 11p15.2 disruption | SOX6 | 11p15.2 |
| Leukodystrophy with 11q14.2-q14.3 |  | 11q14.2q22.3 |
| 12q14.1-q15 Microdeletion | Multiple (LEMD3 and GRIP1 candidate) | 12q14.3 |
| 12q24.21-q24.23 Microduplication | Multiple | 12q24.21-q24.23 |
| Hereditary hemorrhagic telangiectasia, type 2 | ACVRL1 | 12q13.13 |
| Ulnar-mammary | TBX3 | 12q24.21 |
| Buschke-Ollendorff syndrome/Osteopoikilosis, SS and MR | LEMD3 | 12q14.3 |
| Chondrodysplasia | COL2A1 | 12q13.11q13.2 |
| Holt-Oram syndrome | TBX5 | 12q24.21 |
| Noonan syndrome | PTPN11 | 12q24.13 |
| Pallister-Killian | Multiple | 12p |
| Timothy syndrome (TS) | CACNA1C | 12p13.33 |
| 12p11.23 associato a schizofrenia | C12orf11 e PPFIBP1 |  |
| Holoprosencephaly 5 | ZIC2 | 13q32.3 |
| Brachydactyly | GPC5, GPC6 | 13q31.3 |
| Hirschsprung | EDNRB | 13q22.3 |
| Retinoblastoma (RB1) | RB1 | 13q14.2 |
| 14q11.2 Microdeletion | Multiple | 14q11.2 |
|  | CHD8 candidate |  |
|  | SUPT164 candidate |  |
| 14q22-q23 Microdeletion | Multiple | 14q22-q23 |
| 14q12 deletion syndrome | FOXG1B | 14q12 |
| Branchiootic syndrome-3 | SIX1 | 14q23.1 |
| Microphthalmia syn. 6, pituitary hypoplasia (MCOPS6) | SIX6 | 14q22.2q22.3 |
| 15q11 BP1-BP2 interval deletion & duplication |  | 15q11.2 |
| 15q11.2-q12 duplication;reciprocal PWS/AS region |  | 15q11.2q12 |
| 15q21 deletion syndrome |  | 15q21.1q31.2 |
| 15q26.3 deletion |  | 15q26.3 |
| Marfan syndrome (MFS) | FBN1 | 15q21.1 |
| 15q11-q13 Microduplication | Multiple | 15q11-q13 |
| 15q13.3 Microdeletion | Multiple | 15q13.3 |
|  | CHRNA7 candidate |  |
| 15q24.1-q24.3 Microdeletion | Multiple | 15q24.1-q24.3 |
| Angelman | UBE3A | 15q11.2 |
| Congenital diaphragmatic hernia (CDH) | CHD2 | 15q26.1 |
|  | NR2F2 | 15q26.2 |
| Oculocutaneous albinism 2 (OCA2) | OCA2 | 15q13.1 |
| Prader-Willi (PWS) | SNRPN NECDIN snoRNAs | 15q11.2 |
| 16p11.2 Microdeletion | Multiple | 16p11.2 |
| 16p11.2-p12.2 Microdeletion | Multiple | 16p11.2-p12.2 |
| 16p13.1 Microdeletion predisposing to autism and/or mental retardation * | Multiple | 16p13.1 |
| 16p13.3 Microdeletion/Severe Rubinstein-Taybi | CREBBP | 16p13.3 |
|  | DNASE1 |  |
| 16q11.2-q12.2 Microdeletion | Multiple | 16q11.2-q12.2 |
|  | SALL1 candidate |  |
|  | ZNF423 candidate |  |
| Polycystic kidney disease 1 (PKD1) | PKD1 | 16p13.3 |
| Townes-Brocks 1 | SALL1 | 16q12.1 |
| Tuberous sclerosis 2 (TSC2) | TSC2 | 16p13.3 |
| Rubinstein-Taybi (RTS) | CREBBP | 16p13.3 |
| ATR-16 syndrome |  |  |
| 17q21.3 Microdeletion | Multiple | 17q21.3 |
|  | MAPT candidate |  |
| Campomelic dysplasia (CMPD) | SOX9 | 17q24.3 |
| Lissencephaly 1 | PAFAH1B1 (LIS1) | 17p13.3 |
| Miller-Dieker | PAFAH1B1 (LIS1) | 17p13.3 |
| Nephropathic cystinosis | CTNS | 17p13.3 |
| Neurofibromatosis 1 (NF1)/MR | NF1 | 17q11.2 |
| Potocki-Lupski/17p11.2 Microduplication | Multiple | 17p11.2 |
| Renal cysts and diabetes (RCAD) | HNF1B | 17q12 |
| Smith-Magenis (SMS) | RAI1 | 17p11.2 |
| Charcot-Marie-Tooth disease type 1A | PMP22 | 17p12 |
| Cystinosis, Nephropathic (CTNS) | CTNS | 17p13.2p13.3 |
| Hereditary Neuropathy with Liability to Pressure Palsies (HNPP) | PMP22 | 17p11.2 |
| Osteogenesis imperfecta congenita | COL1A1 | 17q21.33 |
| Pitt-Hopkins | TCF4 | 18q21.1 |
| Chromosome 18q deletion syndrome |  | 18q23 |
| Chromosome 18p deletion syndrome |  | 18p11.31 |
| Dyggve-Melchior-Clausen syndrome (DMC) | DYM | 18q21.1 |
| Alagille Sydrome (AGS) | JAG1 | 20p12.2 |
| Brachydactyly, type C (BDC) | GDF5 | 20q11.2 |
| 20p12.2 | PAK7 |  |
| Down syndrome critical region (DSCR) | Multiple | 21q22.13 |
| Holoprosencephaly 1 (HPE1) | TMEM1 | 21q22.3 |
| Early-onset Alzheimer disease with cerebral amyloid angiopathy | Multiple |  |
| 22q11.2 Distal microdeletion | Multiple | 22q11.2 |
| 22q11.21 Microduplication | Multiple (TBX1 candidate) | 22q11.21 |
| 22q13.3 Microdeletion | Multiple (SHANK3 and ARSA candidate) | 22q13.3 |
| Cat-eye | Multiple | 22q11.1 |
| DiGeorge/Velocardiofacial (VCF) | HIRA TBX1 | 22q11.21 |
| Neurofibromatosis 2 (NF2) | NF2 | 22q12.2 |
| Xp11.22-linked mental retardation | Multiple | Xp11.22 |
|  | HSD17B10 candidate |  |
|  | HUWE1 candidate |  |
| Xp11.3 Microdeletion | Multiple | Xp11.3 |
|  | RP2 candidate |  |
|  | ZNF674 candidate |  |
| Xp11.4-p21.2 Contiguous gene deletion | Multiple | Xp11.4-p21.2 |
|  | IL1RAPL1 |  |
|  | OTC |  |
| Adrenal hypoplasia congenita (AHC) | NR0B1 | Xp21.2 |
| Androgen insensitivity | AR | Xq12 |
| Choroideremia | CHM | Xq21.2 |
| Craniofrontonasal | EFNB1 | Xq13.1 |
| FMR1 microdeletion | FMR1 | Xq27.3 |
| Focal dermal hypoplasia/Goltz | PORCN | Xp11.23 |
| Glycerol kinase deficiency (GKD) | GK | Xp21.2 |
| Hemophilia A | F8 | Xq28 |
| Kallmann 1 | KAL1 | Xp22.31 |
| Langer mesomelic dysplasia (LMD) | SHOX | Xpter-Xp22.3 & Ypter-Yp11.32 |
| Leri-Weill dyschondrosteosis (LWD) | SHOX | Xpter-Xp22.3 & Ypter-Yp11.32 |
| Lowe | OCRL | Xq25 |
| Microphthalmia 7 with linear skin defects | Multiple | Xp22.2 |
| Mohr-Tranebjaerg | TIMM8A | Xq22.1 |
| Norrie | NDP | Xp11.3 |
| Opitz | MID1 | Xp22.2 |
| Ornithine transcarbamylase deficiency (OTC) | OTC | Xp11.4 |
| Pelizaeus-Merzbacher | PLP1 | Xq22.2 |
| Simpson-Golabi-Behmel (SGBS) | GPC3 | Xq26.2 |
| Steroid sulfatase deficiency | STS | Xp22.31 |
| X-linked agammaglobulinemia | BTK | Xq22.1 |
| X-linked Alport (ATS) | COL4A5 | Xq22.3 |
| X-linked heterotaxy | ZIC3 | Xq26.3 |
| X-linked idiopathic short stature (ISSX) | SHOX | Xpter-Xp22.3 & Ypter-Yp11.32 |
| X-linked infantile spasms, CDKL5-related | CDKL5 | Xp22.13 |
| X-linked lymphoproliferative (XLP) | SH2D1A | Xq25 |
| X-linked mental retardation 21 | IL1RAPL1 | Xp21.3 |
| X-linked mental retardation with isolated growth hormone deficiency | SOX3 | Xq27.1 |
| X-linked mental retardation with microcephaly & disproportionate pontine and cerebellar hypoplasia | CASK | Xp11.4 |
| Aarskog-Scott | FGD1 | Xp11.22 |
| Adrenoleukodystrophy | ABCD1 | Xq28 |
| ATR-X, and others | ATRX | Xq21.1 |
| Autistic features, X-linked, susceptibility to, 2 | NLGN4 | Xp22.31p22.32 |
| Börjeson-Forssman-Lehmann | PHF6 | Xq26.2 |
| BPNH, OPD | FLNA | Xq28 |
| Cerebellar hypoplasia | OPHN1 | Xq12 |
| Cleft lip/Cleft palate | PHF8 | Xp11.22 |
| Coffin-Lowry | RPS6KA3 | Xp22.12 |
| Cornelia de Lange syndrome 2 (CDLS2) | SMC1L1 | Xp11.22p11.21 |
| Cutis laxa, X-linked/Occipital horn syndrome | ATP7A | Xq12q13 |
| Danon disease | LAMP2 | Xq24 |
| Dent disease | CLCN5 | Xp11.22 |
| Duchenne/Becker mascular dystrophy (DMD/BMD) | DMD | Xp21.1p21.2 |
| Epilepsy | ATP6AP2 | Xp11.4 |
| Epilepsy, macrocephaly | SYN1 | Xp11.23 |
| Fabry disease | GLA | Xq22.1 |
| Golabi-Ito-Hall syndrome | PQBP1 | Xp11.23 |
| Growth hormone deficiency | SOX3 | Xq27.1 |
| HADH2 deficinency | HADH2 | Xp11.22 |
| Hunter | IDS | Xq28 |
| Incontinentia pigmenti (IP) | IKBKG (NEMO) | Xq28 |
| Infantile spasm syndrome, X-linked (ISSX) | ARX | Xp21.3 |
| Infantile spasm syndrome, X-linked (ISSX) | CDKL5 | Xp21.3 |
| Lenz microphthalmia | BCOR | Xp11.4 |
| Lesch-Nyhan syndrome (LNS) | HPRT1 | Xq26.2 |
| Lissencephaly, X-linked (LISX) | DCX | Xq22.3q23 |
| MASA, CRASH, HSAS syndromes | LICAM | Xq28 |
| MECP2 male duplication syndrome | MECP2 | Xq28 |
| Menkes disease (MNK) | ATP7A | Xq12q13 |
| Mental retardation X-linked growth horm. Def | SOX3 | Xq27.1 |
| Monoamine oxidase-A deficiency | MAOA | Xp11.3 |
| Mucopolysaccharidosis, type II (MPS2) | IDS | Xq28 |
| Nance-Horan | NHS | Xp22.13 |
| Orofaciodigital syndrome (OFD1) | OFD1 | Xp22.2 |
| PRPP synthetase hyperactivity | PRPS1 | Xq22.3 |
| Pyruvate dehydrogenase deficiency | PDHA1 | Xp22.12 |
| Renpenning, Sutherland-Haan, Hamel | PQBP1 | Xp11.23 |
| Retinoschisis, X-linked juvinille (RSI) | XLRS1 | Xp22.13 |
| Rett syndrome (RTT); MECP2 deletion | MECP2 | Xq28 |
| Rett-like, infantile spasms | CDKL5 | Xp22.13 |
| Sex reversal, dosage-sensitive (DSS) | NROB1 | Xp21.2 |
| Spermine synthase deficiency | SMS | Xp22.11 |
| Stocco dos Santos X-Linked Mental Retardation | SHROOM4 (KIAA1202) | Xp11.22p11.21 |
| West, Proud, XLAG, Partington syndromes | ARX | Xp21.3 |
| XIST deficiency;usually ring X chromosome | XIST | Xq13.2 |
| X-linked chronic granulomatous disease | CYBB | Xp11.4 |
| X-Linked Cornelia De Lange syndrome | SMC1L1 | Xp11.22 |
| X-linked dyskeratosis congenita | DKC1 | Xq28 |
| X-linked hypohidrotic ectodermal dysplasia | EDA | Xq13.1 |
| X-linked hypophosphatemic rickets | PHEX | Xp22.11 |
| X-linked lymphoproliferative syndrome (XLP) | SH2D1A | Xq25 |
| X-Linked Mental Retardation (XLMR) | NXF5 | Xq22.1 |
| X-Linked Mental Retardation (XLMR) | VCX3A | Xp22.31 |
| X-Linked Mental Retardation (XLMR) | AP1S2 | Xp22.2 |
| X-Linked Mental Retardation (XLMR) | TM4SF2 (TSPAN7) | Xp11.4 |
| X-Linked Mental Retardation (XLMR) | ZNF674 | Xp11.3 |
| X-Linked Mental Retardation (XLMR) | ZNF41 | Xp11.3 |
| X-Linked Mental Retardation (XLMR) | ELK1 | Xp11.23 |
| X-Linked Mental Retardation (XLMR) | ZNF81 | Xp11.23 |
| X-Linked Mental Retardation (XLMR) | SLC38A5 | Xp11.23 |
| X-Linked Mental Retardation (XLMR) | FTSJ1 | Xp11.23 |
| X-Linked Mental Retardation (XLMR) | JARID1C | Xp11.22 |
| X-Linked Mental Retardation (XLMR) | KLF8 | Xp11.21 |
| X-Linked Mental Retardation (XLMR) | DLG3 | Xq13.1 |
| X-Linked Mental Retardation (XLMR) | ZNF261 (ZMYM3) | Xq13.3 |
| X-Linked Mental Retardation (XLMR) | KIAA2022 | Xq13.3 |
| X-Linked Mental Retardation (XLMR) | ZDHHC15 | Xq13.3 |
| X-Linked Mental Retardation (XLMR) | ACSL4 | Xq22.3 |
| X-Linked Mental Retardation (XLMR) | PAK3 | Xq22.3 |
| X-Linked Mental Retardation (XLMR) | AGTR2 | Xq23 |
| X-Linked Mental Retardation (XLMR) | CUL4B | Xq24 |
| X-Linked Mental Retardation (XLMR) | GRIA3 | Xq25 |
| X-Linked Mental Retardation (XLMR) | ZDHHC9 | Xq25 |
| X-Linked Mental Retardation (XLMR) | RPS6KA3 | Xp22.12 |
| SRY Dosage Abnormalities | SRY | Yp11.31 |
| X-linked idiopathic short stature (ISSX) | SHOX | Xpter-Xp22.3 & Ypter-Yp11.32 |
| XX male | SRY | Yp11.31 |
| XY gonadal dysgenesis | SRY | Yp11.31 |
