# Supplementary material for: Enhancer Chip: Detecting Human Copy Number Variations in Regulatory Elements
Source: PLoS One. 2012 Dec 20;7(12):e52264. doi: 10.1371/journal.pone.0052264 (PMC3527541; doi:10.1371/journal.pone.0052264)
Supplement: Table S3 — VISTA enhancer loci localized in DVG variations. (DOC) [file pone.0052264.s004.doc]

**Supplementary Table S3 -** VISTA enhancer loci localized in *DVG* variations

| **Enhancer ID** | **Enhancer position** | **Enhancer Bracketing Genes** | **Enhancer Expression** | **Variation Position** | **Variation ID** |
| --- | --- | --- | --- | --- | --- |
|  |  |  |  |  |  |
| hs705 | chr1:3190581-3191428 | PRDM16(intragenic) | Positive | chr1:3127184-3226660 | 4194 |
|  |  |  |  | chr1:3152593-3333816 | 4195 |
| hs289 | chr1:10732070-10733118 | FLJ20321(intragenic) | Positive | chr1:10673194-10796866 | 53188 |
|  |  |  |  | chr1:10677562-10795161 | 114819 |
|  |  |  |  | chr1:10704811-10859919 | 4205 |
|  |  |  |  | chr1:10719645-10750432 | 9872 |
|  |  |  |  | chr1:10731782-10745649 | 109977 |
|  | chr1:10757664-10758631 chr1:10781239-10781744 | FLJ20321-FLJ37118 | Negative Positive | chr1:10673194-10796866 | 53188 |
| hs361 |  |  |  | chr1:10677562-10795161 | 114819 |
| hs389 |  |  |  | chr1:10704811-10859919 | 4205 |
|  |  |  |  | chr1:10742502-11055147 | 3282 |
| hs408 | chr1:10851570-10852173 | FLJ20321-FLJ37118 | Positive | chr1:10704811-10859919 | 4205 |
|  |  |  |  | chr1:10742502-11055147 | 3282 |
| hs417 | chr1:10925202-10925728 chr1:10965129-10966144 | FLJ20321-FLJ37118 | Negative | chr1:10742502-11055147 | 3282 |
| hs241 |  |  | Positive |  |  |
| hs850 | chr1:39291500-39292441 | POU3F1-RRAGC | Negative | chr1:39269692-39339253 | 84182 |
| hs442 | chr1:83345366-83346271 | LPHN2-FLJ23033 | Positive | chr1:83328562-84008311 | 3297 |
| hs443 | chr1:83360512-83361298 | LPHN2-FLJ23033 | Negative | chr1:83328562-84008311 | 3297 |
| hs444 | chr1:83411289-83412040 | LPHN2-FLJ23033 | Negative | chr1:83328562-84008311 | 3297 |
|  |  |  |  | chr1:83400337-83564026 | 4233 |
| hs445 | chr1:83878319-83879217 | LPHN2-FLJ23033 | Negative | chr1:83328562-84008311 | 3297 |
|  |  |  |  | chr1:83585288-83950632 | 74549 |
|  |  |  |  | chr1:83598248-83955219 | 64085 |
|  |  |  |  | chr1:83603357-83944228 | 2313 |
|  |  |  |  | chr1:83604512-83929788 | 97424 |
|  |  |  |  | chr1:83846248-83883528 | 84339 |
| hs1672 | chr1:113540056-113542020 | SLC16A1-LRIG2 | Positive | chr1:113157135-116741372 | 3306 |
|  |  |  |  | chr1:113246630-116699268 | 2322 |
|  |  |  |  | chr1:113375437-113852815 | 69591 |
| hs201 | chr1:163939700-163940637 | CDCA1-PBX1 | Negative | chr1:163902256-164063835 | 4260 |
| hs202 | chr1:164023244-164024214 |  |  |  |  |
| hs1442 | chr1:169910396-169913079 | KIFAP3(intragenic) | Positive | chr1:169883302-169924707 | 84649 |
| hs734 | chr1:217766122-217767351 | GPATC2(intragenic) | Negative | chr1:217634630-217788033 | 4270 |
| hs1257 | chr1:218207432-218208498 | SPATA17-CGI-115 | Positive | chr1:218181128-218215541 | 399 |
|  |  |  |  | chr1:218181128-218215541 | 34774 |
| hs1714 | chr1:232753930-232757436 | SIPA1L2-KIAA1383 | Negative | chr1:232595015-233379219 | 8348 |
| hs826 | chr10:23490838-23491508 | PTF1A-C10orf67 | Negative | chr10:23440604-23505846 | 112633 |
| hs1567 | chr10:35972892-35975932 | FZD8-ANKRD30A | Positive | chr10:35871949-35974254 | 112654 |
| hs727 | chr10:60791178-60792149 | TFAM-PHYHIPL | Negative | chr10:60718033-60971361 | 3806 |
| hs595 | chr10:76177765-76179000 | ADK(intragenic) | Positive | chr10:76007903-76373904 | 48630 |
| hs484 | chr10:77164564-77165639 | ZNF503-C10orf11 | Positive | chr10:77141491-77178843 | 112737 |
| hs795 | chr10:77683451-77684636 | C10orf11(intragenic) | Negative | chr10:77420559-77770449 | 3811 |
|  |  |  |  | chr10:77536046-77736622 | 2879 |
|  |  |  |  | chr10:77544669-77701506 | 30511 |
|  |  |  |  | chr10:77555491-77736622 | 8661 |
| hs320 | chr10:77726861-77727618 | C10orf11(intragenic) | Positive | chr10:77420559-77770449 | 3811 |
|  |  |  |  | chr10:77536046-77736622 | 2879 |
|  |  |  |  | chr10:77555491-77736622 | 8661 |
| hs1491 | chr10:80295449-80297087 | RPS24-ZMIZ1 | Positive | chr10:80266999-80314300 | 53395 |
| hs1006 | chr10:102244842-102246334 | WNT8B-SEC31B | Positive | chr10:102151850-102380635 | 3818 |
|  |  |  |  | chr10:102203071-102275642 | 2888 |
|  |  |  |  | chr10:102220965-102269005 | 30514 |
|  |  |  |  | chr10:102221424-102269206 | 9645 |
|  |  |  |  | chr10:102222957-102275642 | 8665 |
| hs224 | chr10:102372214-102374206 | HIF1AN-PAX2 | Negative | chr10:102151850-102380635 | 3818 |
| hs225 | chr10:102374886-102375478 |  |  |  |  |
| hs285 | chr10:102414915-102415578 | HIF1AN-PAX2 | Negative | chr10:102378636-102565736 | 3819 |
| hs286 | chr10:102419119-102419731 |  |  |  |  |
| hs228 | chr10:102447081-102448985 |  |  |  |  |
| hs229 | chr10:102546590-102548095 | PAX2(intragenic) | Positive |  |  |
| hs520 | chr10:102551472-102552372 |  | Negative |  |  |
| hs939 | chr10:103094612-103095386 | LBX1-BTRC | Negative | chr10:103054982-103452645 | 8666 |
| hs897 | chr10:103245010-103246610 | BTRC(intragenic) | Negative |  |  |
| hs326 | chr10:103266649-103267972 | BTRC(intragenic) | Positive |  |  |
| hs513 | chr10:103540180-103541030 | FGF8-NPM3 | Positive | chr10:103529485-103577142 | 112798 |
| hs841 | chr10:118854124-118855243 | KIAA1598-VAX1 | Positive | chr10:118851383-118854960 | 85491 |
| hs935 | chr10:119310483-119311458 | EMX2-RAB11FIP2 | Positive | chr10:119285669-119315906 | 112825 |
| hs1221 | chr10:119313709-119314860 |  | Negative |  |  |
| hs771 | chr10:125108319-125109394 | BUB3-GPR26 | Negative | chr10:125057217-125269159 | 3824 |
| hs578 | chr10:125188987-125190075 | BUB3-GPR26 | Positive | chr10:125057217-125269159 | 3824 |
|  |  |  |  | chr10:125174371-125278806 | 47960 |
| hs542 | chr10:129964063-129965094 | MKI67-MGMT | Negative | chr10:129870293-130361981 | 47892 |
| hs562 | chr10:131106522-131108742 | MKI67-MGMT | Positive | chr10:131107941-131208744 | 48464 |
| hs232 | chr10:131691086-131692848 | DKFZp667B0210(intragenic) | Positive | chr10:131610723-131808881 | 4716 |
| hs1488 | chr11:1985072-1987372 | MRPL23-IGF2 | Positive | chr11:1857112-2057646 | 29893 |
|  |  |  |  | chr11:1915273-1999138 | 37117 |
|  |  |  |  | chr11:1948961-2014709 | 29894 |
|  |  |  |  | chr11:1948961-2014709 | 48763 |
|  |  |  |  | chr11:1985127-2057646 | 29891 |
| hs863 | chr11:31502035-31503157 | IMMP1L(intragenic) | Positive | chr11:31062955-31547932 | 9180 |
| hs234 | chr11:31685357-31686884 | ELP4(intragenic) | Positive | chr11:31624927-31689819 | 112978 |
| hs1082 | chr11:31816452-31818421 | PAX6(intragenic) | Positive | chr11:31815588-31852830 | 112980 |
| hs866 | chr11:32052398-32053496 | PAX6-RCN1 | Negative | chr11:32009541-32189124 | 4734 |
| hs577 | chr11:32808120-32809049 | CCDC73(intragenic) | Negative | chr11:32714643-33049940 | 3843 |
|  |  |  |  | chr11:32792784-32958609 | 2915 |
|  |  |  |  | chr11:32796092-33004024 | 8685 |
| hs561 | chr12:16869033-16871415 | LMO3-FLJ22655 | Negative | chr12:16791659-16950747 | 113271 |
| hs661 | chr12:16940708-16942322 |  | Positive |  |  |
| hs993 | chr12:17311784-17313759 | LMO3-FLJ22655 | Positive | chr12:17217902-17395321 | 4770 |
| hs895 | chr12:24233884-24235634 | SOX5-FLJ32894 | Positive | chr12:24164088-24313719 | 48013 |
| hs233 | chr12:24291012-24292724 | SOX5(intragenic) | Negative |  |  |
| hs1468 | chr12:59361630-59364518 | LRIG3-SLC16A7 | Positive | chr12:59350361-59367360 | 35105 |
| hs614 | chr13:63681506-63682317 | PCDH20-PCDH9 | Negative | chr13:63281939-63721248 | 3016 |
| hs882 | chr13:71533037-71534195 | KLHL1-DACH1 | Positive | chr13:71378041-71542952 | 4810 |
| hs182 | chr13:72255836-72257039 | DACH1(intragenic) | Negative | chr13:72079800-72269554 | 4811 |
|  |  |  |  | chr13:72091863-72287614 | 29631 |
| hs340 | chr13:72256090-72256838 | DACH1(intragenic) | Negative | chr13:72079800-72269554 | 4811 |
|  |  |  |  | chr13:72091863-72287614 | 29631 |
| hs619 | chr13:72333516-72334988 | DACH1(intragenic) | Positive | chr13:72328325-72485405 | 3912 |
| hs188 | chr13:72345980-72348230 |  |  |  |  |
| hs138 | chr13:72425787-72428335 |  |  |  |  |
| hs796 | chr13:95313852-95315441 | GPR180-SOX21 | Positive | chr13:94906537-95392644 | 47869 |
| hs488 | chr13:95358263-95360017 | GPR180-SOX21 | Positive | chr13:94906537-95392644 | 47869 |
|  |  |  |  | chr13:95358502-95368070 | 113600 |
| hs1246 | chr13:112268067-112269088 | C13orf16-SOX1 | Positive | chr13:112074854-112353994 | 3926 |
| hs571 | chr13:112793153-112794130 | SOX1-C13orf28 | Negative | chr13:112688182-112795607 | 113630 |
| hs428 | chr14:26377372-26378803 | STXBP6-NOVA1 | Positive | chr14:26227880-26405118 | 3931 |
| hs736 | chr14:28036097-28036839 | NOVA1-FOXG1B | Negative | chr14:27839000-28053462 | 30654 |
| hs1075 | chr14:28398563-28400621 | NOVA1-FOXG1B | Negative | chr14:28065099-28443242 | 3932 |
| hs944 | chr14:29613303-29614728 | FOXG1B-PRKD1 | Negative | chr14:29511827-29698386 | 175 |
| hs566 | chr14:29684896-29686744 |  | Positive |  |  |
| hs791 | chr14:30989066-30990943 | PRKD1-KIAA1333 | Negative | chr14:30800223-31911745 | 7031 |
| hs1647 | chr14:32940050-32944094 | AKAP6(intragenic) | Negative | chr14:32858480-33016442 | 3933 |
| hs345 | chr14:34068299-34069069 | NPAS3(intragenic) | Negative | chr14:33835920-34190494 | 47870 |
| hs346 | chr14:34122328-34123032 |  |  |  |  |
| hs1676 | chr14:34385301-34386834 | NPAS3-EGLN3 | Negative | chr14:34232772-34629603 | 47862 |
| hs1486 | chr14:95836414-95839481 | CLMN-C14orf49 | Positive | chr14:95758559-96141595 | 8775 |
| hs910 | chr15:33091527-33092659 | GREM1-RYR3 | Negative | chr15:31982066-34867298 | 7058 |
|  |  |  |  | chr15:32798938-33221215 | 5324 |
| hs830 | chr15:38159507-38161007 | MEIS2-TMCO5 | Positive | chr15:38115684-38192456 | 47987 |
| hs660 | chr15:67198974-67200134 | SMAD6-SMAD3 | Positive | chr15:67185932-67273894 | 5433 |
| hs359 | chr15:70391576-70392613 | TLE3-UACA | Positive | chr15:70387199-70393247 | 113985 |
|  |  |  |  | chr15:70387774-70391690 | 72272 |
| hs1510 | chr15:74870847-74872794 | ARID3B(intragenic) | Positive | chr15:74364360-75569130 | 37170 |
| hs1494 | chr15:82179952-82181888 | STARD5-RKHD3 | Negative | chr15:81941520-82224069 | 3971 |
| hs90 | chr16:6548841-6550064 | A2BP1(intragenic) | Negative | chr16:6139140-6713777 | 30003 |
|  |  |  |  | chr16:6399837-6701566 | 30004 |
| hs88 | chr16:7418980-7419830 | A2BP1(intragenic) | Negative | chr16:7405611-7471606 | 49756 |
| hs91 | chr16:7431988-7433156 |  |  |  |  |
| hs93 | chr16:8133046-8134289 | A2BP1-MGC2654 | Negative | chr16:8112808-8138100 | 35414 |
| hs1242 | chr16:10319829-10320876 | GRIN2A-ATF7IP2 | Positive | chr16:10008309-10415517 | 5328 |
| hs95 | chr16:17096348-17097253 | NOMO3-XYLT1 | Negative | chr16:16917137-18780937 | 8814 |
| hs96 | chr16:17745104-17746413 | XYLT1-NOMO2 | Negative | chr16:16917137-18780937 | 8814 |
|  |  |  |  | chr16:17534100-19083035 | 3112 |
| hs96 | chr16:17745104-17746413 | XYLT1-NOMO2 | Negative | chr16:17573099-19159306 | 3994 |
|  |  |  |  | chr16:17592162-18264088 | 8815 |
|  |  |  |  | chr16:17600805-18208068 | 114130 |
|  |  |  |  | chr16:17609474-18165043 | 9756 |
| hs97 | chr16:18240361-18241096 | XYLT1-NOMO2 | Negative | chr16:16917137-18780937 | 8814 |
|  |  |  |  | chr16:17534100-19083035 | 3112 |
|  |  |  |  | chr16:17573099-19159306 | 3994 |
|  |  |  |  | chr16:17592162-18264088 | 8815 |
| hs98 | chr16:22684122-22685282 | CDR2-HS3ST2 | Negative | chr16:21577815-22687850 | 481 |
|  |  |  |  | chr16:21592738-22708328 | 29493 |
|  |  |  |  | chr16:21594011-22705302 | 29492 |
|  |  |  |  | chr16:21594442-22712501 | 110100 |
|  |  |  |  | chr16:22294124-22772309 | 3996 |
|  |  |  |  | chr16:22405811-22723664 | 3115 |
|  |  |  |  | chr16:22444844-22710691 | 72508 |
|  |  |  |  | chr16:22448197-22725739 | 7096 |
|  |  |  |  | chr16:22480336-22686022 | 793 |
|  |  |  |  | chr16:22480336-22686022 | 2207 |
|  |  |  |  | chr16:22536473-22706547 | 4934 |
|  |  |  |  | chr16:22543733-22713596 | 105526 |
|  |  |  |  | chr16:22543733-22713596 | 114137 |
|  |  |  |  | chr16:22548207-22715492 | 30765 |
|  |  |  |  | chr16:22551908-22712190 | 66956 |
|  |  |  |  | chr16:22557932-22704521 | 37845 |
|  |  |  |  | chr16:22558786-22709680 | 32104 |
|  |  |  |  | chr16:22605878-22712781 | 5434 |
|  |  |  |  | chr16:22622551-22709831 | 102229 |
|  |  |  |  | chr16:22625768-22712703 | 58755 |
|  |  |  |  | chr16:22626385-22763352 | 323 |
|  |  |  |  | chr16:22628912-22710590 | 114138 |
|  |  |  |  | chr16:22673854-22777777 | 29661 |
|  |  |  |  | chr16:22673854-22744032 | 29663 |
|  |  |  |  | chr16:22673855-22777777 | 53723 |
| hs99 | chr16:25759215-25760529 | HS3ST4(intragenic) | Negative | chr16:25624711-25811254 | 3998 |
| hs124 | chr16:49094466-49095242 | N4BP1-CBLN1 | Positive | chr16:49061753-49129705 | 325 |
| hs69 | chr16:51757191-51758243 | SALL1-CHD9 | Negative | chr16:51752051-51763930 | 49719 |
| hs65 | chr16:51958288-51959688 | SALL1-CHD9 | Positive | chr16:51878565-52316530 | 47872 |
| hs154 | chr16:52242610-52244077 |  | Negative |  |  |
| hs55 | chr16:54092614-54094121 | KIAA1005-IRX3 | Negative | chr16:54079661-54234378 | 4955 |
| hs167 | chr16:54132651-54133957 |  | Negative |  |  |
| hs53 | chr16:54139806-54140268 |  | Negative |  |  |
| hs52 | chr16:54143854-54144867 |  | Positive |  |  |
| hs51 | chr16:54323420-54324336 | IRX3-IRX5 | Positive | chr16:54312083-54327501 | 114180 |
| hs46 | chr16:54523175-54524067 | IRX3-IRX5 | Negative | chr16:54456599-54567234 | 9277 |
| hs47 | chr16:54540302-54541446 |  | Negative |  |  |
| hs43 | chr16:54575878-54577925 | IRX3-IRX5 | Positive | chr16:54564282-54752209 | 4006 |
| hs42 | chr16:54579726-54580655 |  | Negative |  |  |
| hs41 | chr16:54611467-54612437 |  | Negative |  |  |
| hs44 | chr16:54616212-54617037 |  | Negative |  |  |
| hs39 | chr16:54618981-54620264 |  | Negative |  |  |
| hs38 | chr16:54624504-54625510 |  | Negative |  |  |
| hs37 | chr16:54650598-54651882 |  | Positive |  |  |
| hs36 | chr16:54657182-54658317 |  | Negative |  |  |
| hs35 | chr16:54665329-54667258 |  | Negative |  |  |
| hs34 | chr16:54679467-54680359 |  | Negative |  |  |
| hs32 | chr16:54784643-54785615 | IRX3-IRX5 | Negative | chr16:54753168-54847382 | 9278 |
| hs31 | chr16:54799102-54800160 |  |  |  |  |
| hs25 | chr16:55240593-55241874 | IRX5-IRX6 | Negative | chr16:55236480-55387532 | 4956 |
| hs151 | chr16:55329289-55330760 |  | Positive |  |  |
| hs102 | chr16:59629741-59631254 | GOT2-CDH8 | Negative | chr16:59617082-59700873 | 114203 |
| hs104 | chr16:61243163-61244434 | GOT2-CDH8 | Negative | chr16:61115741-61267738 | 4960 |
| hs106 | chr16:64651540-64652894 | CDH8-CDH11 | Negative | chr16:64650301-64651918 | 88174 |
| hs22 | chr16:72254566-72255825 | PMFBP1-ATBF1 | Positive | chr16:71976344-72274347 | 4011 |
| hs14 | chr16:78387021-78388685 chr16:78397855-78398920 | WWOX(intragenic) | Negative | chr16:78291173-78417314 | 3128 |
| hs163 |  |  |  | chr16:78359016-78411757 | 490 |
| hs14 | chr16:78387021-78388685 chr16:78397855-78398920 | WWOX(intragenic) | Negative | chr16:78359016-78411757 | 35411 |
| hs163 |  |  |  | chr16:78370716-78404257 | 35412 |
| hs9 | chr16:78933253-78934686 | WWOX(intragenic) | Negative | chr16:78872859-79131738 | 47873 |
| hs7 | chr16:79026563-79028162 | WWOX(intragenic) | Negative | chr16:78872859-79131738 | 47873 |
|  |  |  |  | chr16:79006863-79126670 | 47894 |
|  |  |  |  | chr16:79026434-79039202 | 67051 |
| hs4 | chr16:80372593-80373755 | MAF-DNCL2B | Positive | chr16:80341745-80549683 | 4968 |
| hs3 | chr16:80423343-80424652 |  | Negative |  |  |
| hs2 | chr16:85620095-85621736 | MGC22001-KIAA0182 | Negative | chr16:85618564-85637882 | 110108 |
| hs845 | chr17:12375783-12376586 | MAP2K4-MYOCD | Negative | chr17:12339578-12745298 | 47896 |
|  |  |  |  | chr17:12343840-12378185 | 35428 |
|  |  |  |  | chr17:12350887-12381764 | 35430 |
| hs362 | chr17:35011848-35012557 | FLJ22578-LHX1 | Positive | chr17:34435487-35081387 | 8843 |
|  |  |  |  | chr17:34814328-36297053 | 37189 |
|  |  |  |  | chr17:34875147-35040221 | 2223 |
|  |  |  |  | chr17:34944593-35030559 | 3143 |
|  |  |  |  | chr17:34962048-35147394 | 4032 |
| hs849 | chr17:35057889-35058730 | MRM1-LHX1 | Negative | chr17:34435487-35081387 | 8843 |
|  |  |  |  | chr17:34814328-36297053 | 37189 |
|  |  |  |  | chr17:34962048-35147394 | 4032 |
| hs363 hs364 | chr17:35329349-35329944 chr17:35336093-35337226 | AATF(intragenic) | Positive | chr17:34814328-36297053 | 37189 |
|  |  |  | Negative | chr17:35166406-35381639 | 5001 |
| hs177 | chr17:35447270-35448478 | ACACA(intragenic) | Negative | chr17:34814328-36297053 | 37189 |
| hs819 | chr17:35474054-35475155 |  | Negative |  |  |
| hs627 | chr17:37774485-37774988 | NEUROD2-PPP1R1B | Positive | chr17:37761921-37788355 | 77591 |
| hs730 | chr17:41640235-41640900 | ETV4-MEOX1 | Negative | chr17:41508997-41689929 | 5007 |
| hs729 | chr17:41668736-41669927 |  |  |  |  |
| hs933 | chr17:46682250-46682768 | HOXB6-HOXB7 | Negative | chr17:46602673-46836837 | 4040 |
|  |  |  |  | chr17:46620402-46698710 | 53230 |
| hs370 | chr18:34480079-34481120 | KIAA1328(intragenic) | Negative | chr18:34110763-34481867 | 3165 |
| hs371 | chr18:35063482-35064528 | BRUNOL4(intragenic) | Positive | chr18:34946002-35128390 | 5052 |
| hs372 | chr18:35178249-35179130 | BRUNOL4-PIK3C3 | Negative | chr18:35096468-35277549 | 47853 |
| hs797 | chr18:37498660-37499574 | BRUNOL4-PIK3C3 | Negative | chr18:37482117-37649307 | 4057 |
| hs375 | chr18:44770271-44771284 | IER3IP1-SMAD2 | Negative | chr18:44762014-44801196 | 114583 |
| hs1397 | chr18:56471647-56474866 | MALT1-ZNF532 | Positive | chr18:56472630-56479136 | 110197 |
| hs634 | chr18:76006820-76008476 | GALR1-SALL3 | Positive | chr18:75973397-76008096 | 77959 |
|  |  |  |  | chr18:75973397-76008096 | 88958 |
|  |  |  |  | chr18:75992039-76008096 | 88960 |
| hs1083 | chr18:76064036-76065306 | GALR1-SALL3 | Negative | chr18:76024385-76277832 | 4064 |
| hs1141 | chr18:76070697-76071834 | GALR1-SALL3 | Negative | chr18:76024385-76277832 | 4064 |
|  |  |  |  | chr18:76068686-76072576 | 5926 |
| hs587 | chr19:30379565-30380645 | CCNE1-C19orf2 | Negative | chr19:30272050-30441498 | 114780 |
|  |  |  |  | chr19:30369625-30395547 | 35622 |
|  |  |  |  | chr19:30379221-30396469 | 35623 |
| hs381 | chr19:30494614-30495503 | C19orf2(intragenic) | Positive | chr19:30465196-30546184 | 50126 |
| hs721 | chr19:30574387-30575360 | C19orf2-ZNF536 | Positive | chr19:30572756-30580597 | 9793 |
| hs1496 | chr2:5549618-5553025 | ADI1-SOX11 | Positive | chr2:5497669-5668354 | 3343 |
| hs1276 | chr2:19740634-19741760 | OSR1-TTC32 | Negative | chr2:19734958-19781658 | 35776 |
| hs1273 | chr2:19774115-19776218 |  | Positive |  |  |
| hs1348 | chr2:50840428-50844037 | NRXN1-ASB3 | Positive | chr2:50798461-50861049 | 2383 |
| hs1076 | chr2:58695819-58697323 | FANCL-BCL11A | Negative | chr2:58394543-61340450 chr2:58398297-61282740 chr2:58410949-61295473 | 2386 3361 8373 |
| hs1174 | chr2:58748340-58750140 |  | Positive |  |  |
| hs1012 | chr2:58770304-58771290 |  | Negative |  |  |
| hs1071 | chr2:58799729-58800607 |  | Positive |  |  |
| hs1018 | chr2:58809796-58811611 |  | Negative |  |  |
| hs1090 | chr2:58811475-58812905 |  | Negative |  |  |
| hs1063 | chr2:58857680-58858956 |  | Negative |  |  |
| hs1152 | chr2:58859997-58861674 |  | Positive |  |  |
| hs1232 | chr2:58891096-58892548 |  | Negative |  |  |
| hs1113 | chr2:58948607-58950015 |  | Negative |  |  |
| hs1067 | chr2:58975738-58977115 |  | Positive |  |  |
| hs1199 | chr2:59102071-59103380 |  | Positive |  |  |
| hs391 | chr2:59108123-59108845 |  | Negative |  |  |
| hs392 | chr2:59133026-59134590 |  | Negative |  |  |
| hs1196 | chr2:59161996-59164461 |  | Negative |  |  |
| hs1181 | chr2:59178992-59180242 |  | Positive |  |  |
| hs393 | chr2:59198905-59200529 |  | Positive |  |  |
| hs1077 | chr2:59202276-59203399 |  | Negative |  |  |
| hs1154 | chr2:59203217-59204548 |  | Negative |  |  |
| hs1143 | chr2:59303870-59305029 |  | Negative |  |  |
| hs975 | chr2:59304974-59306893 |  | Positive |  |  |
| hs1119 | chr2:59476604-59477955 | FANCL-BCL11A | Positive | chr2:58394543-61340450 chr2:58398297-61282740 chr2:58410949-61295473 | 2386 3361 8373 |
| hs836 | chr2:59540640-59541937 |  | Positive |  |  |
| hs394 | chr2:59746377-59746992 |  | Positive |  |  |
| hs1057 | chr2:59888700-59891476 |  | Negative |  |  |
| hs1209 | chr2:59894793-59896957 |  | Negative |  |  |
| hs1204 | chr2:59896819-59898978 |  | Negative |  |  |
| hs395 | chr2:59947272-59948294 |  | Negative |  |  |
| hs1021 | chr2:59998338-59999656 |  | Negative |  |  |
| hs1164 | chr2:60005531-60007545 |  | Negative |  |  |
| hs1072 | chr2:60055628-60056970 |  | Negative |  |  |
| hs822 | chr2:60077064-60078749 |  | Negative |  |  |
| hs396 | chr2:60137953-60139763 |  | Negative |  |  |
| hs1120 | chr2:60223849-60225179 |  | Negative |  |  |
| hs397 | chr2:60297377-60299041 |  | Negative |  |  |
| hs946 | chr2:60317429-60318194 |  | Negative |  |  |
| hs779 | chr2:60352514-60353602 |  | Positive |  |  |
| hs399 | chr2:60441495-60442515 |  | Positive |  |  |
| hs1535 | chr2:60498057-60502013 |  | Positive |  |  |
| hs1111 | chr2:60516097-60518092 |  | Negative |  |  |
| hs1176 | chr2:60794812-60796264 | BCL11A-PAPOLG | Negative |  |  |
| hs1142 | chr2:60855056-60856888 |  | Positive |  |  |
| hs1229 | chr2:66397542-66398943 | FLJ16124-MEIS1 | Negative | chr2:66329056-66499865 | 3363 |
| hs831 | chr2:67281257-67282540 | MEIS1-ETAA1 | Positive | chr2:67266707-67337017 | 2389 |
|  |  |  |  | chr2:67273120-67316250 | 8949 |
| hs1584 | chr2:67414599-67416139 | MEIS1-ETAA1 | Negative | chr2:67287279-67465662 | 3364 |
| hs1565 | chr2:67427102-67431497 | MEIS1-ETAA1 | Negative | chr2:67287279-67465662 | 3364 |
|  |  |  |  | chr2:67424193-67438040 | 9414 |
| hs1140 | chr2:104648312-104650249 | FLJ30294-POU3F3 | Negative | chr2:104593605-104760102 | 3380 |
| hs1303 | chr2:104667872-104670648 |  | Positive |  |  |
| hs1554 | chr2:104686690-104688638 |  | Positive |  |  |
| hs401 | chr2:104736518-104737365 |  | Positive |  |  |
| hs1121 | chr2:105452973-105454169 | FLJ30294-POU3F3 | Negative | chr2:105437715-105468193 | 78676 |
|  |  |  |  | chr2:105440021-105501404 | 115427 |
| hs803 | chr2:156399791-156401487 | KCNJ3-NR4A2 | Negative | chr2:156296807-156467237 | 270 |
| hs580 | chr2:156403022-156404115 |  | Positive |  |  |
| hs551 | chr2:156584380-156586345 | KCNJ3-NR4A2 | Negative | chr2:156436900-156599346 | 3398 |
| hs691 | chr2:156762038-156763191 | KCNJ3-NR4A2 | Negative | chr2:156751921-156775966 | 9966 |
| hs1477 | chr2:159779317-159780341 | LOC92196-TANC1 | Positive | chr2:159651734-159953466 | 53156 |
| hs640 | chr2:164574007-164575458 | FIGN(intragenic) | Positive | chr2:164503649-164683626 | 4315 |
| hs606 | chr2:164657051-164658028 | FIGN-GRB14 | Negative |  |  |
| hs420 | chr2:164661606-164662430 |  | Negative |  |  |
| hs246 | chr2:176940070-176941410 | KIAA1715-HOXD13 | Positive | chr2:176921920-177080951 | 115536 |
| hs1207 | chr2:213141079-213142308 | ERBB4(intragenic) | Negative | chr2:213120523-213165247 | 53484 |
| hs504 | chr2:220271494-220272006 | DNPEP-DES | Negative | chr2:220206919-220390543 | 30 |
| hs493 | chr2:223079383-223080439 | PAX3(intragenic) | Negative | chr2:222928927-223105072 | 4328 |
| hs921 | chr2:236962599-236964857 | CENTG2(intragenic) | Positive | chr2:236872105-237083077 | 8399 |
|  |  |  |  | chr2:236952464-237203359 | 7254 |
| hs249 | chr2:237071364-237071848 | CENTG2-GBX2 | Positive | chr2:236872105-237083077 | 8399 |
|  |  |  |  | chr2:236952464-237203359 | 7254 |
|  |  |  |  | chr2:237015987-237155577 | 115611 |
|  |  |  |  | chr2:237071306-237081163 | 115612 |
| hs1243 | chr2:237165066-237166143 | ASB18-IQCA | Negative | chr2:236952464-237203359 | 7254 |
| hs992 | chr20:38862746-38864025 | DHX35-MAFB | Negative | chr20:38797871-38974947 | 5137 |
| hs1592 | chr20:39461549-39463625 | MAFB-TOP1 | Negative | chr20:39462401-39463626 | 79195 |
| hs1101 | chr20:39472648-39473702 | MAFB-TOP1 | Negative | chr20:39465750-39477857 | 110407 |
| hs1692 | chr21:17968630-17971417 | C21orf34(intragenic) | Negative | chr21:17967386-18142224 | 5159 |
| hs726 | chr21:19814334-19815155 | PRSS7-NCAM2 | Negative | chr21:19407812-22372729 | 9325 |
| hs497 | chr22:19741204-19741707 | GP1BB-TBX1 | Negative | chr22:19019088-21053198 | 31071 |
|  |  |  |  | chr22:19423250-19766782 | 8901 |
|  |  |  |  | chr22:19678592-20311988 | 115055 |
|  |  |  |  | chr22:19699493-19785073 | 115056 |
|  |  |  |  | chr22:19717320-19802779 | 30150 |
|  |  |  |  | chr22:19741202-19755688 | 115058 |
| hs515 | chr22:19749356-19750055 | TBX1(intragenic) | Positive | chr22:19019088-21053198 | 31071 |
|  |  |  |  | chr22:19423250-19766782 | 8901 |
|  |  |  |  | chr22:19678592-20311988 | 115055 |
|  |  |  |  | chr22:19699493-19785073 | 115056 |
|  |  |  |  | chr22:19717320-19802779 | 30150 |
| hs515 | chr22:19749356-19750055 | TBX1(intragenic) | Positive | chr22:19741202-19755688 | 115058 |
|  |  |  |  | chr22:19748994-19752621 | 115059 |
| hs496 | chr22:38392037-38392599 | SOX10-PICK1 | Negative | chr22:38388465-38549017 | 5185 |
| hs486 | chr22:38394345-38395199 | SOX10-PRKCABP | Positive |  |  |
| hs491 | chr22:38429240-38430515 | SOX10-PICK1 | Positive |  |  |
| hs492 | chr22:38442140-38442879 | SOX10-PICK1 | Positive |  |  |
| hs490 | chr22:38446392-38446997 | SOX10-PICK1 | Negative |  |  |
| hs1472 | chr3:55524543-55526260 | WNT5A-ERC2 | Positive | chr3:55490629-55665793 | 3430 |
| hs997 | chr3:71499477-71500970 | FOXP1(intragenic) | Negative | chr3:71477946-71508865 |  |
|  |  |  |  |  | 79793 |
| hs973 | chr3:71507469-71508874 |  |  |  | 91352 |
|  |  |  |  |  |  |
| hs843 | chr3:137638911-137640108 | SOX14-CLDN18 | Positive | chr3:137608870-137644253 | 32519 |
|  |  |  |  | chr3:137611789-137933835 | 3449 |
| hs1043 | chr3:147125146-147126336 | ZIC4-ZIC1 | Positive | chr3:147099081-147146462 | 115865 |
| hs1509 | chr4:53823605-53826463 | SCFD2(intragenic) | Negative | chr4:53818092-53834394 | 92102 |
| hs1651 | chr4:56919589-56923793 | CEP135-AASDH | Positive | chr4:56923506-56925017 | 98716 |
| hs1374 | chr4:90917033-90921053 | MMRN1-MGC48628 | Positive | chr4:90866819-90952424 | 51489 |
|  |  |  |  | chr4:90879613-91439573 | 29746 |
| hs918 | chr4:109062224-109063783 | LEF1(intragenic) | Negative | chr4:109059609-109065625 | 80420 |
| hs507 | chr4:111530174-111530773 | ENPEP-PITX2 | Negative | chr4:111516352-111570589 | 92311 |
|  |  |  |  | chr4:111517645-111538602 | 92312 |
| hs1582 | chr4:124383428-124386454 | SPRY1-ANKRD50 | Positive | chr4:124211226-125379347 chr4:124238947-125253161 chr4:124260091-125255527 chr4:124271321-125247395 | 3512 |
|  |  |  |  |  | 2543 |
| hs1525 | chr4:124775814-124779530 |  |  |  | 31186 |
|  |  |  |  |  | 8454 |
| hs1361 | chr4:153287655-153290517 | FBXW7-TMEM154 | Positive | chr4:153256133-153309538 | 51274 |
| hs1327 | chr5:2204457-2208380 | IRX4-IRX2 | Positive | chr5:2208323-2211665 | 104854 |
|  |  |  |  |  | 111148 |
| hs215 | chr5:50335050-50336643 | PARP8-ISL1 | Positive | chr5:50327032-50355888 | 36416 |
| hs1432 | chr5:81318882-81321683 | ATG10(intragenic) | Positive | chr5:81165704-81408744 | 8490 |
|  |  |  |  | chr5:81210225-81514737 | 3560 |
| hs429 | chr5:88179457-88179988 | MEF2C-CETN3 | Negative | chr5:88164521-88204505 | 93139 |
| hs789 | chr5:88673410-88674494 | MEF2C-CETN3 | Positive | chr5:88669984-88860383 | 4460 |
| hs191 | chr5:91036888-91038899 | ARRDC3-NR2F1 | Positive | chr5:90955522-91135272 | 4461 |
| hs1170 | chr5:92526066-92527353 | ARRDC3-NR2F1 | Positive | chr5:92515026-92738896 chr5:92518295-92703611 | 4462 4463 |
|  |  |  |  |  |  |
| hs1222 | chr5:92536353-92537380 |  | Negative |  |  |
|  |  |  |  |  |  |
| hs1550 | chr5:92741502-92744743 | ARRDC3-NR2F1 | Negative | chr5:92703902-92847345 | 4464 |
| hs546 | chr5:92811465-92813217 |  | Positive |  |  |
| hs273 | chr5:93649881-93651432 | DKFZP564D172-MGC34713 | Negative | chr5:93593894-93664537 | 111263 |
|  |  |  |  | chr5:93643170-93789309 | 51756 |
| hs1733 | chr5:106813861-106814679 | EFNA5(intragenic) | Negative | chr5:106720912-107110506 | 47882 |
| hs1594 | chr5:134355508-134357399 | CATSPER3-PITX1 | Negative | chr5:134353751-134406800 | 111333 |
|  |  |  |  | chr5:134356321-134379057 | 111334 |
| hs1366 | chr6:38358690-38360084 | BTBD9(intragenic) | Positive | chr6:38291830-38390679 | 77 |
| hs1225 | chr6:41380559-41381515 | NCR2-FOXP4 | Negative | chr6:41379626-41391470 | 105620 |
| hs787 | chr6:50552252-50554393 | DEFB112-TFAP2D | Negative | chr6:50519339-50667841 | 3612 |
| hs538 | chr6:51515894-51517015 | PKHD1(intragenic) | Negative | chr6:51516913-51518431 | 99536 |
| hs1054 | chr6:98261649-98263117 | C6orf167-POU3F2 | Negative | chr6:98242041-98263613 | 52033 |
| hs576 | chr6:100657912-100659391 | MCHR2-SIM1 | Positive | chr6:100257279-100675050 | 8525 |
| hs1302 | chr6:112822313-112826325 | LOC442247-MARCKS | Positive | chr6:112810569-112849366 | 93962 |
| hs293 | chr7:1265154-1266318 | LOC90637-MICAL-L2 | Positive | chr7:1092493-1278992 | 4519 |
|  |  |  |  | chr7:1191689-1363454 | 30248 |
|  |  |  |  | chr7:1199095-1689947 | 3660 |
|  |  |  |  | chr7:1259427-1306398 | 111775 |
|  |  |  |  | chr7:1261466-1266858 | 105667 |
| hs173 | chr7:1267832-1269169 | LOC90637-MICAL-L2 | Negative | chr7:1092493-1278992 | 4519 |
|  |  |  |  | chr7:1191689-1363454 | 30248 |
|  |  |  |  | chr7:1199095-1689947 | 3660 |
|  |  |  |  | chr7:1259427-1306398 | 111775 |
| hs799 | chr7:9271308-9272358 | NXPH1-NDUFA4 | Positive | chr7:9257996-9274493 | 9558 |
| hs749 | chr7:13450920-13451719 | ARL4A-ETV1 | Positive | chr7:13438942-14554888 | 292 |
| hs550 | chr7:13506207-13507276 |  |  |  |  |
| hs816 | chr7:14379627-14380740 | DGKB(intragenic) | Positive |  |  |
| hs644 | chr7:18885971-18887230 | HDAC9-TWIST1 | Positive | chr7:18730767-18906456 | 94 |
| hs239 | chr7:20829390-20830407 | SP8-SP4 | Negative | chr7:20809566-20844134 | 111823 |
| hs844 | chr7:20832628-20833902 | SP8-SP4 | Positive | chr7:20809566-20844134 | 111823 |
| hs501 | chr7:27249858-27251057 | HOXA13-EVX1 | Negative | chr7:27119294-27295010 | 111829 |
| hs629 | chr7:27288268-27289491 | EVX1-HIBADH | Positive |  |  |
| hs465 | chr7:35590614-35591629 | TBX20-FLJ22313 | Negative | chr7:35542153-35726804 | 719 |
| hs298 | chr7:96633582-96634303 | SHFM1-DLX5 | Positive | chr7:96620279-96669211 | 111934 |
| hs1418 | chr7:155264047-155265809 | EN2-PRR8 | Positive | chr7:155124154-155307408 | 112046 |
| hs508 | chr8:11604182-11604695 | GATA4(intragenic) | Positive | chr8:7219660-12005446 | 37303 |
|  |  |  |  | chr8:7426605-12450985 | 37304 |
|  |  |  |  | chr8:7437436-12456008 | 37305 |
|  |  |  |  | chr8:7451668-12441708 | 37306 |
|  |  |  |  | chr8:7460958-12435737 | 37307 |
|  |  |  |  | chr8:7897445-12444758 | 37311 |
|  |  |  |  | chr8:7901538-12444659 | 37312 |
|  |  |  |  | chr8:7903571-12438688 | 37313 |
|  |  |  |  | chr8:7906494-12437216 | 37314 |
|  |  |  |  | chr8:7993421-12424508 | 37315 |
|  |  |  |  | chr8:7993425-12429396 | 37316 |
|  |  |  |  | chr8:8024528-12061148 | 37317 |
|  |  |  |  | chr8:8024710-12055996 | 37318 |
|  |  |  |  | chr8:8098045-12324868 | 37319 |
|  |  |  |  | chr8:8100604-12316276 | 37320 |
|  |  |  |  | chr8:8113868-12306378 | 37321 |
| hs231 | chr8:25775160-25776669 | EBF2-PPP2R2A | Negative | chr8:25699652-25801382 | 7679 |
| hs1718 | chr8:33660674-33665591 | DUSP26-UNC5D | Negative | chr8:33414494-33882742 | 9090 |
| hs303 | chr8:66148927-66149836 | CYP7B1-ARMC1 | Negative | chr8:65988830-66163053 | 4598 |
|  |  |  |  | chr8:65989146-66300480 | 8605 |
| hs1318 | chr8:77598007-77600645 | HNF4G-ZFHX4 | Positive | chr8:77568431-77621596 | 95509 |
| hs909 | chr8:100680409-100681809 | VPS13B-COX6C | Positive | chr8:100404151-100838624 | 3744 |
| hs1400 | chr8:134681048-134683216 | ST3GAL1-ZFAT1 | Negative | chr8:134638338-134827078 | 4610 |
| hs558 | chr8:144613510-144614569 | ZC3H3(intragenic) | Negative | chr8:144557035-144649192 | 30298 |
|  |  |  |  | chr8:144559165-144615195 | 53408 |
| hs628 | chr9:159657-160780 | APOA1- | Positive | chr9:19847-223670 | 95777 |
|  |  |  |  | chr9:31994-383816 | 3753 |
|  |  |  |  | chr9:40910-317370 | 8616 |
|  |  |  |  | chr9:40910-499586 | 8617 |
|  |  |  |  | chr9:40910-354913 | 34455 |
|  |  |  |  | chr9:46587-272191 | 52810 |
|  |  |  |  | chr9:46587-398673 | 52811 |
|  |  |  |  | chr9:48710-209354 | 65299 |
|  |  |  |  | chr9:57964-362209 | 2780 |
|  |  |  |  | chr9:63212-322740 | 31461 |
|  |  |  |  | chr9:149481-274606 | 37771 |
| hs304 | chr9:8095553-8096166 | JMJD2C-PTPRD | Positive | chr9:7887756-8418897 | 33070 |
| hs307 | chr9:16710536-16711184 | BNC2(intragenic) | Positive | chr9:16125443-20690244 | 52762 |
|  |  |  |  | chr9:16641966-16809203 | 3763 |
| hs529 | chr9:17322200-17324371 | C9orf39(intragenic) | Positive | chr9:16125443-20690244 | 52762 |
| hs308 | chr9:17331971-17332580 |  | Negative |  |  |
| hs1390 | chr9:38425496-38429775 | IGFBPL1-FLJ35740 | Positive | chr9:38335475-38436992 | 47939 |
|  |  |  |  | chr9:38409006-38684425 | 33092 |
| hs1395 | chr9:71422469-71425428 | FAM122A-PRKACG | Negative | chr9:71234845-71663340 | 5262 |
| hs312 | chr9:81471747-81473114 | PSAT1-LOC401531 | Positive | chr9:81429309-81474084 | 2825 |
|  |  |  |  | chr9:81434812-81473308 | 96143 |
| hs974 | chr9:83412700-83413972 | TLE4-TLE1 | Positive | chr9:83367633-84907654 | 305 |
| hs1228 | chr9:83711693-83712782 |  | Negative |  |  |
| hs240 | chr9:83727123-83728378 |  | Positive |  |  |
| hs722 | chr9:84009516-84010866 |  | Positive |  |  |
| hs1359 | chr9:84264195-84266950 | TLE1(intragenic) | Positive |  |  |
| hs556 | chr9:84299210-84300117 | TLE1(intragenic) | Positive | chr9:83367633-84907654 | 305 |
|  |  |  |  | chr9:84299110-84300567 | 100682 |
| hs583 | chr9:84438198-84439339 | TLE1-FLJ46321 | Negative | chr9:83367633-84907654 | 305 |
| hs1323 | chr9:87604796-87606909 | NTRK2-AGTPBP1 | Negative | chr9:86511916-88451262 | 29497 |
| hs1339 | chr9:92292484-92293889 | GADD45G-DIRAS2 | Positive | chr9:91963403-92343382 | 65451 |
|  |  |  |  | chr9:92160163-92338734 | 760 |
| hs1258 | chr9:98257859-98259405 | PTCH1(intragenic) | Positive | chr9:98239963-98326547 | 112438 |
|  |  |  |  | chr9:98245375-98283861 | 112439 |
| hs1030 | chr9:128516934-128518372 | PBX3(intragenic) | Positive | chr9:128477788-128554741 | 112500 |
| hs818 | chr9:128520992-128522653 | PBX3(intragenic) | Positive |  |  |
| hs983 | chr9:128525348-128527214 | PBX3(intragenic) | Negative |  |  |
| hs316 | chr9:128583956-128584480 | PBX3(intragenic) | Negative | chr9:128565431-128584660 | 36914 |
|  |  |  |  | chr9:128565976-128591974 | 36915 |
|  |  |  |  | chr9:128574974-128591515 | 36917 |
| hs1015 | chr9:128919674-128920432 | PBX3-FAM125B | Positive | chr9:128875957-129036958 | 3781 |
| hs1108 | chr9:128945054-128946417 | PBX3-FAM125B | Negative | chr9:128875957-129036958 | 3781 |
|  |  |  |  | chr9:128944285-128954309 | 37917 |
| hs1328 | chr9:133353647-133355097 | ASS1(intragenic) | Positive | chr9:133350106-133357957 | 105844 |
| hs876 | chr9:133540555-133541228 | PRDM12(intragenic) | Positive | chr9:133512543-133573304 | 112526 |
|  |  |  |  | chr9:133534345-133541511 | 112527 |
| hs122 | chrX:25017067-25018756 | POLA-ARX | Positive | chrX:25016288-25024878 | 96556 |
| hs145 | chrX:25018871-25020532 | POLA-ARX | Positive | chrX:81968292-81984217 | 96797 |
| hs554 | chrX:81978920-81979942 | SH3BGRL-POU3F4 | Negative | chrX:81975442-81981942 | 96796 |
|  |  |  |  | chrX:81977171-81991626 | 37062 |
|  |  |  |  | chrX:81979342-81984217 | 96800 |
| hs537 | chrX:124653246-124654824 | ODZ1-WDR40C | Negative | chrX:124453577-124942286 | 8914 |
| hs597 | chrX:137064390-137065532 | ZIC3-FGF13 | Negative | chrX:136983479-137458872 | 7764 |
| hs427 | chrX:139169379-139171545 | ATP11C-SOX3 | Positive | chrX:139061154-139298500 | 37351 |
|  |  |  |  | chrX:139156206-139220679 | 103286 |
| hs588 | chrX:139674499-139675403 | SOX3-CDR1 | Negative | chrX:139617542-139675085 | 7765 |
