# Supplementary material for: Enhancer Chip: Detecting Human Copy Number Variations in Regulatory Elements
Source: PLoS One. 2012 Dec 20;7(12):e52264. doi: 10.1371/journal.pone.0052264 (PMC3527541; doi:10.1371/journal.pone.0052264)
Supplement: Table S4 — VISTA enhancer loci localized in Decipher Syndromes Regions. (DOC) [file pone.0052264.s005.doc]

**Supplementary Table S4 -** VISTA enhancer loci localized in Decipher Syndromes Regions

| **Enhancer ID** | **Enhancer position** | **Enhancer Bracketing Genes** | **Enhancer Expression** | **Decipher Syndrome Position** | | **Decipher Syndrome** |
| --- | --- | --- | --- | --- | --- | --- |
|  |  |  |  |  |  |  |
| hs1082 | chr11:31816452-31818421 | Pax6 (intragenic) | Positive | chr11:31803509-32510988 | | WAGR 11p13  deletion syndrome |
| hs855 | chr11:31989173-31990022 | Rcn1-Pax6os1 | Positive |  |  |  |
| hs866 | chr11:32052398-32053496 |  | Negative |  |  |  |
| hs113 | chr11:32197490-32198734 | 0610012H03Rik (intragenic) | Positive |  |  |  |
| hs1498 | chr12:67127104-67129730 | Grip1 (intragenic) | Positive | chr12:65071919-68645525 | | 12q14  microdeletion syndrome |
| hs1510 | chr15:74870847-74872794 | Arid3b (intragenic) |  | chr15:74377174-76162277 | | 15q24  recurrent microdeletion syndrome |
| hs98 | chr16:22684122-22685282 | Otoa-Hs3st2 | Negative | chr16:21613956-29042192 | | 16p11.2-p12.2  microdeletion syndrome |
| hs99 | chr16:25759215-25760529 | Zkscan2-4930571K23Rik |  |  |  |  |
| hs100 | chr16:26226210-26227347 |  |  |  |  |  |
| hs1445 | chr17:2079772-2081236 | Smg6 (intragenic) | Positive | chr17:1-2545429 | | Miller-Dieker syndrome (MDS) |
| hs924 | chr17:2081998-2084289 |  |  |  |  |  |
| hs362 | chr17:35011848-35012557 | Lhx1-Mrm1 | Positive | chr17:34907366-36076803 | | RCAD  (renal cysts and diabetes) |
| hs849 | chr17:35057889-35058730 |  | Negative |  |  |  |
| hs363 | chr17:35329349-35329944 | Aatf (intragenic) | Positive |  |  |  |
| hs364 | chr17:35336093-35337226 |  | Negative |  |  |  |
| hs177 | chr17:35447270-35448478 |  | Negative |  |  |  |
| hs819 | chr17:35474054-35475155 |  | Negative |  |  |  |
| hs390 | chr2:57972375-57973175 | Gm12070-Ccdc85a | Negative | chr2:57741796-61738334 | 2p15-16.1  microdeletion syndrome | |
| hs937 | chr2:58146107-58147489 | Vrk2-Gm12070 | Negative |  |  |  |
| hs1076 | chr2:58695819-58697323 | 5730522E02Rik (intragenic) | Negative |  |  |  |
| hs1174 | chr2:58748340-58750140 |  | Positive |  |  |  |
| hs1012 | chr2:58770304-58771290 |  | Negative |  |  |  |
| hs1071 | chr2:58799729-58800607 |  | Positive |  |  |  |
| hs1018 | chr2:58809796-58811611 |  | Negative |  |  |  |
| hs1090 | chr2:58811475-58812905 |  | Negative |  |  |  |
| hs1063 | chr2:58857680-58858956 |  | Negative |  |  |  |
| hs1152 | chr2:58859997-58861674 |  | Positive |  |  |  |
| hs1232 | chr2:58891096-58892548 |  | Negative |  |  |  |
| hs1113 | chr2:58948607-58950015 |  | Negative |  |  |  |
| hs1067 | chr2:58975738-58977115 |  | Positive |  |  |  |
| hs1199 | chr2:59102071-59103380 |  | Positive |  |  |  |
| hs391 | chr2:59108123-59108845 | 5730522E02Rik (intragenic) | Negative |  |  | |
| hs392 | chr2:59133026-59134590 |  | Negative |  |  |  |
| hs1196 | chr2:59161996-59164461 |  | Negative |  |  |  |
| hs1181 | chr2:59178992-59180242 |  | Positive | chr2:57741796-61738334 | | 2p15-16.1  microdeletion syndrome |
| hs393 | chr2:59198905-59200529 |  | Positive |  |  |  |
| hs1077 | chr2:59202276-59203399 |  | Negative |  |  |  |
| hs1154 | chr2:59203217-59204548 |  | Negative |  |  |  |
| hs1143 | chr2:59303870-59305029 | Bcl11a-5730522E02Rik | Negative |  |  |  |
| hs975 | chr2:59304974-59306893 |  | Positive |  |  |  |
| hs1119 | chr2:59476604-59477955 |  | Positive |  |  |  |
| hs836 | chr2:59540640-59541937 |  | Positive |  |  |  |
| hs394 | chr2:59746377-59746992 |  | Positive |  |  |  |
| hs1057 | chr2:59888700-59891476 |  | Negative |  |  |  |
| hs1209 | chr2:59894793-59896957 |  | Negative |  |  |  |
| hs1204 | chr2:59896819-59898978 |  | Negative |  |  |  |
| hs395 | chr2:59947272-59948294 |  | Negative |  |  |  |
| hs1021 | chr2:59998338-59999656 |  | Negative |  |  |  |
| hs1164 | chr2:60005531-60007545 |  | Negative |  |  |  |
| hs1072 | chr2:60055628-60056970 |  | Negative |  |  |  |
| hs822 | chr2:60077064-60078749 |  | Negative |  |  |  |
| hs396 | chr2:60137953-60139763 |  | Negative |  |  |  |
| hs1120 | chr2:60223849-60225179 |  | Negative |  |  |  |
| hs397 | chr2:60297377-60299041 |  | Negative |  |  |  |
| hs946 | chr2:60317429-60318194 |  | Negative |  |  |  |
| hs779 | chr2:60352514-60353602 |  | Positive |  |  |  |
| hs399 | chr2:60441495-60442515 |  | Positive |  |  |  |
| hs1535 | chr2:60498057-60502013 |  | Positive |  |  |  |
| hs1111 | chr2:60516097-60518092 |  | Negative |  |  |  |
| hs1176 | chr2:60794812-60796264 | Papolg-Bcl11a | Negative |  |  |  |
| hs1142 | chr2:60855056-60856888 |  | Positive |  |  |  |
|  |  |  |  |  | |  |
| hs1253 | chr2:199226864-199228567 | Plcl1-Hsfy2 | Negative | chr2:196925089-205206940 | | 2q33.1 deletion syndrome |
| hs950 | chr2:199681972-199683281 |  | Negative |  |  |  |
| hs497 | chr22:19741204-19741707 | Tbx1-Gp1bb | Negative | chr2:196925089-205206940 | | 2q33.1 deletion syndrome |
| hs515 | chr22:19749356-19750055 | Tbx1(intragenic) | Positive | chr2:239954693-243102476 | | 2q37 monosomy |
| hs633 | chr5:2112055-2113430 | Irx2-Irx4 | Positive | chr5:10001-11723854 | | Cri du Chat Syndrome (5p deletion) |
| hs1327 | chr5:2204457-2208380 |  | Positive |  |  |  |
| hs900 | chr5:3179614-3180432 | Irx1-Irx2 | Negative |  |  |  |
| hs603 | chr5:3182218-3183271 |  | Positive |  |  |  |
| hs533 | chr5:3186439-3187926 |  | Positive |  |  |  |
| hs754 | chr5:3197865-3198942 |  | Positive |  |  |  |
| hs180 | chr5:3226121-3227484 |  | Negative |  |  |  |
| hs261 | chr5:3511978-3513399 |  | Positive |  |  |  |
| hs1642 | chr7:95874847-95878101 | Slc25a13(intragenic) | Positive | chr7:95533860-96779486 | | Split hand/foot malformation 1 (SHFM1) |
| hs298 | chr7:96633582-96634303 | Dlx6os1(intragenic) | Positive |  |  |  |
| hs508 | chr8:11604182-11604695 | Gata4(intragenic) | Positive | chr8:8119295-11765719 | | 8p23.1 deletion syndrome |
