# Supplementary material for: Enhancer Chip: Detecting Human Copy Number Variations in Regulatory Elements
Source: PLoS One. 2012 Dec 20;7(12):e52264. doi: 10.1371/journal.pone.0052264 (PMC3527541; doi:10.1371/journal.pone.0052264)
Supplement: Table S5 — CNVs of uncertain significance detected by Enhancer Chip. (DOC) [file pone.0052264.s006.doc]

**Supplementary** **Table S5 -** CNVs of uncertain significance detected by *Enhancer Chip*

|  |  |  |  | **5' Breakpoint** | | **3' Breakpoint** | | **Lenght (bp)** | |
| --- | --- | --- | --- | --- | --- | --- | --- | --- | --- |
| **Sample** | **Sex** | **Chromosome Region** | **Probes** | **from** | **to** | **from** | **to** | **min** | **max** |
| 17 | F | chr11:4813710-4907501 | 7 | 4798659 | 4813909 | 4907359 | 4917349 | 93450 | 118690 |
| 21 | F | chr1:1567790-1666621 | 8 | 1567195 | 1567990 | 1666480 | 1770610 | 98490 | 203415 |
